# Conquering the Host: Determinants of Pathogenesis Learned from Murine Gammaherpesvirus 68

**DOI:** 10.1146/annurev-virology-011921-082615

**Published:** 2021-09-29

**Authors:** Yiping Wang, Scott A. Tibbetts, Laurie T. Krug

**Affiliations:** 1 Department of Molecular Genetics and Microbiology, UF Health Cancer Center, College of Medicine, University of Florida, Gainesville, Florida 32610, USA; 2 HIV and AIDS Malignancy Branch, Center for Cancer Research, National Cancer Institute, Bethesda, Maryland 20892, USA

**Keywords:** gammaherpesvirus, latency, reactivation, pathogenesis, oncogenesis, virus-host interactions

## Abstract

Gammaherpesviruses are an important class of oncogenic pathogens that are exquisitely evolved to their respective hosts. As such, the human gammaherpesviruses Epstein-Barr virus (EBV) and Kaposi sarcoma herpesvirus (KSHV) do not naturally infect nonhuman primates or rodents. There is a clear need to fully explore mechanisms of gammaherpesvirus pathogenesis, host control, and immune evasion in the host. A gammaherpesvirus pathogen isolated from murid rodents was first reported in 1980; 40 years later, murine gammaherpesvirus 68 (MHV68, MuHV-4, *γ*HV68) infection of laboratory mice is a well-established pathogenesis system recognized for its utility in applying state-of-the-art approaches to investigate virus-host interactions ranging from the whole host to the individual cell. Here, we highlight recent advancements in our understanding of the processes by which MHV68 colonizes the host and drives disease. Lessons that inform KSHV and EBV pathogenesis and provide future avenues for novel interventions against infection and virus-associated cancers are emphasized.

## 1. INTRODUCTION

Murine gammaherpesvirus 68 (MHV68, MuHV-4, *γ*HV68), a natural pathogen of murid rodents, is widely used to discover and understand key pathogenic determinants of in vivo gammaherpesvirus infections. MHV68 was isolated from bank voles; related strains have been isolated from bank voles and yellow-necked mice, and numerous rodent species appear to serve as reservoirs (reviewed in 1). MHV68 infection of *Mus musculus* generates a higher viral load but similar course of infection as compared to bank voles (2), providing a powerful system for systematic examination of in vivo gammaherpesvirus infection.

**Figure 1:**
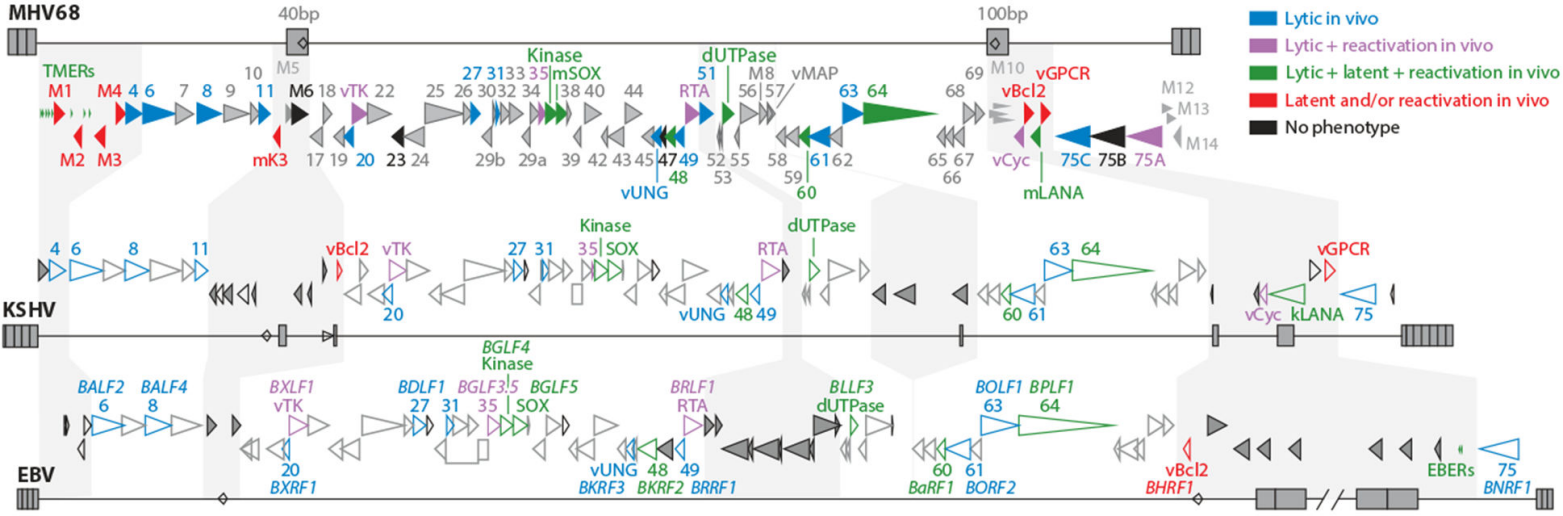
Functional annotation of MHV68 genes and genomic alignment with KSHV and EBV. Diagrams are based on annotations and coordinates in GenBank: U97553.2 (MHV68 strain WUMS), U75698 (human herpesvirus 8 BC-1), and NC_007605 (EBV strain B95-8). Terminal repeats and internal repeats are indicated by gray boxes. Open reading frames considered likely to encode expressed proteins are indicated by triangles that are oriented to show their direction of transcription. Interspersed between core conserved genes are shaded blocks for genes that are unique to each virus (designated M genes for MHV68) or found only in the rhadinovirus (represented by MHV68 and KSHV) or gammaherpesviruses (MHV68, KSHV, and EBV). Colors indicate the broad role of genes in vivo/mice based on phenotypes of viruses with specific mutations in those genes. Detailed descriptions of putative gene functions and mutant phenotypes are available in Supplemental Tables 1 and 2. Abbreviations: EBER, EBV encoded small RNA; EBV, Epstein-Barr virus; kLANA, KSHV latency-associated nuclear antigen; KSHV, Kaposi sarcoma herpesvirus; MHV68, murine gammaherpesvirus 68; mLANA, MHV68 latency-associated nuclear antigen; RTA, replication and transcription activator; TMER, transfer RNA-microRNA-encoded RNA; vCyc, v-cyclin; vGPCR, viral G protein–coupled receptor. Figure adapted with permission from Reference 151.

The MHV68 genome (3) is colinear with Kaposi sarcoma herpesvirus (KSHV, HHV-8) and thus is classified as a member of the *Rhadinovirus* genus with the formal designation of murid gammaherpesvirus 4 (MuHV-4, MuGHV-4) (Figure 1). MHV68 shares key biologic properties with the human gammaherpesviruses KSHV and Epstein-Barr virus (EBV, HHV-4), including establishment of latency in B cells and propensity to drive tumorigenesis. Wild-type MHV68 grows productively in culture and establishes lifelong infection in inbred laboratory and wild mice. Through orchestrated infection and trafficking of multiple cell types, MHV68 traverses mucosal surfaces to access the B cell compartment and disseminate through the host. In secondary lymphoid organs, MHV68 protein products and noncoding RNAs (ncRNAs) engage and usurp germinal center (GC) reactions to gain access to the long-lived memory B cell compartment. As is the case for KSHV and EBV, the coevolutionary virus-host balance typically results in asymptomatic infection, with the oncogenic potential of MHV68 manifesting when host immune control is compromised.

**Figure 2:**
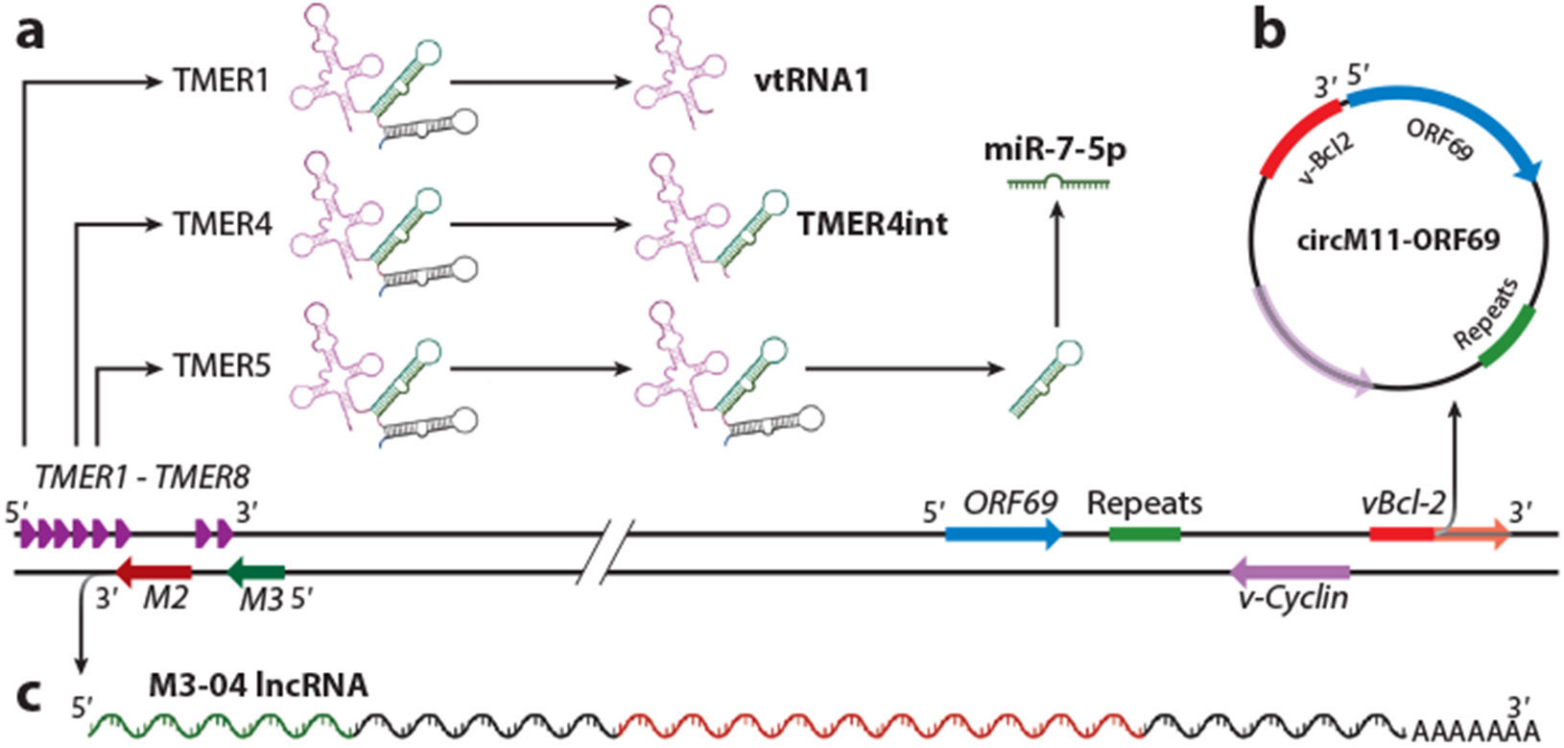
Overview of MHV68 ncRNA types (see sidebar titled Noncoding RNAs). (*a*) MHV68 encodes eight TMER molecules that are expressed as full-length RNAs or intermediate RNAs and are processed to transfer RNA-like molecules (vtRNAs) and microRNAs. The image depicts specific TMER products with published in vivo phenotypes. (*b*) MHV68 encodes at least one circRNA. circRNA is generated by a backsplice, a noncanonical splicing event wherein a downstream donor is spliced to an upstream acceptor. The image depicts the location and orientation of genomic sequences within the circM11-ORF69 RNA. The backsplice is generated from a site within vBcl-2 to just upstream of ORF69. (*c*) MHV68 encodes at least 25 lncRNAs. The image depicts the location and orientation of transcript *M3-04*, which directly overlaps the M3 and M2 ORFs. Abbreviations: circ, circular; lncRNA, long noncoding RNA; MHV68, murine gammaherpesvirus 68; ncRNA, noncoding RNA; ORF, open reading frame; TMER, transfer RNA-microRNA-encoded RNA; vtRNA, viral tRNA. Figure adapted from images created with BioRender.com.

Of the 79 MHV68 open reading frames (ORFs) identified in initial studies (3), 64 and 59 have direct homologs in KSHV and EBV, respectively (Figure 1). The remaining ORFs are unique to MHV68, but several display functional overlap with human gammaherpesvirus proteins that hijack host signaling and evade immune control. Recent genome-wide transcript structure resolution revealed 258 transcript isoforms, including at least 55 new or previously unappreciated ORFs (4). MHV68 encodes numerous types of ncRNAs (see sidebar titled Noncoding RNAs, Figure 2). The majority of ORFs and ncRNAs have been queried for their requirement for virus replication in cell culture and pathogenesis in mice. Summaries and details of the mutant virus phenotypes are provided in Supplemental Tables 1 and 2, respectively.

The ability to generate high titer stocks, thereby enabling synchronous infections, makes MHV68 ideal to mechanistically explore virus-host interactions in culture. Recent discoveries of MHV68 subversion of cellular processes to promote viral gene expression and evade host responses (5-10) have direct implications for KSHV and EBV. The added power of the MHV68 system lies in the ability to query the role of both virus and host factors in the whole animal using sophisticated genetic approaches and cutting-edge technologies. Mutant viruses are generated using bacterial artificial chromosome (BAC)–based recombineering; host factors are evaluated in knockout, bone marrow chimeric and transgenic mice; and complementary virus-host platforms allow precise assessment of gene functions in specific cell types or phases of infection. Such in vivo studies have revealed numerous MHV68 gene products to be multifunctional factors evolved for cell-specific and stage-specific roles in pathogenesis. This complexity explains why many gammaherpesvirus genes are nonessential in cell lines; in vivo analysis is necessary to fully characterize gene functions. Thus, this review primarily focuses on new insights into virus and host determinants required for MHV68 infection and pathogenesis, with an emphasis on those that inform the pathobiology of human gammaherpesviruses.

## 2. PHASES OF THE MHV68 LIFE CYCLE IN VIVO

**Figure 3:**
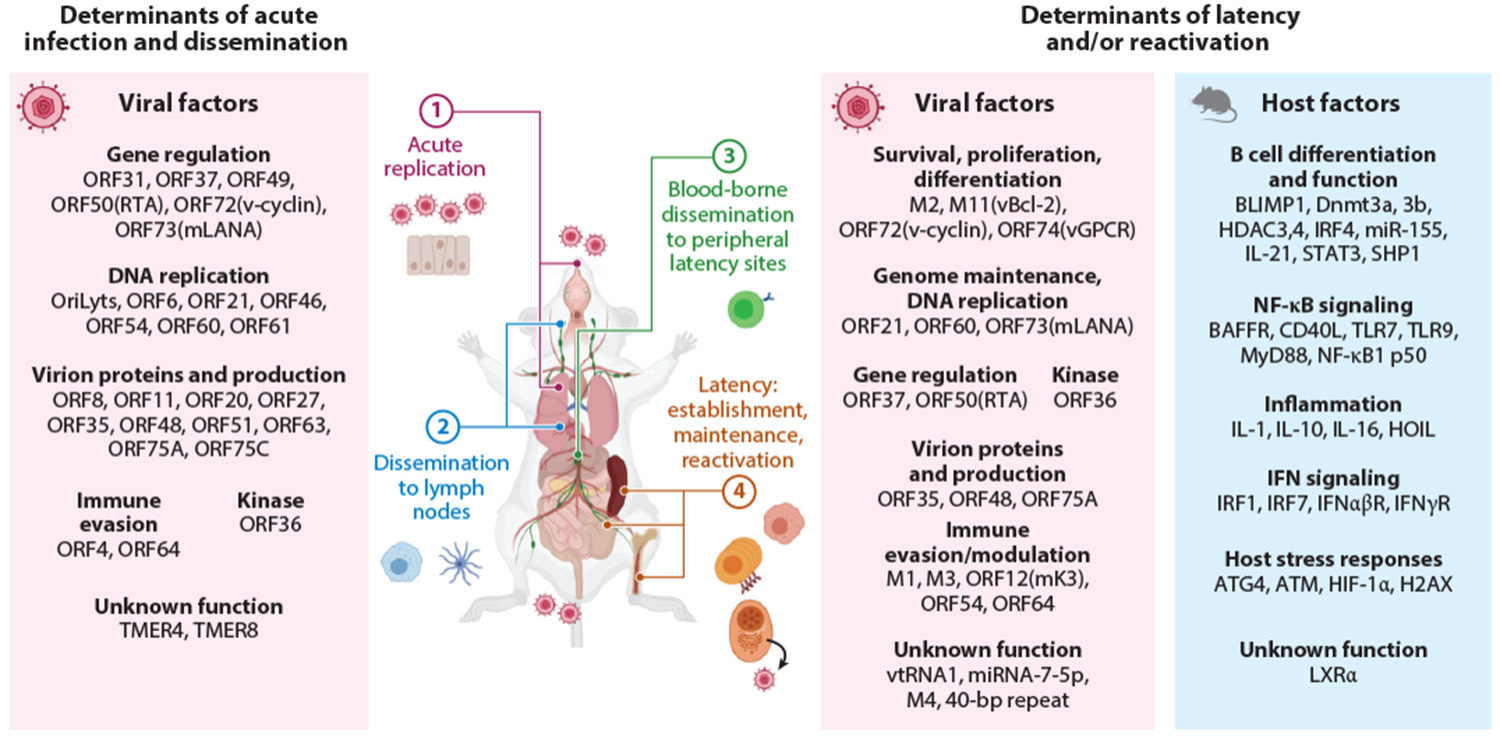
Overview of the MHV68 course of infection. ① Airway inoculation of mice with MHV68 leads to a 4- to 12-day course of acute replication in the lung (with anesthesia) or in the nose (without anesthesia). ② MHV68 traffics via myeloid cells to the draining lymph nodes, either to the mediastinal nodes subsequent to replication in the lungs or to the superficial cervical nodes following replication in the nasal cavity, prior to ③ engagement of B cells for dissemination to peripheral lymphoid tissues. ④ By 16 days post infection, the virus has initiated the establishment of latency in B cells of the spleen and other lymphoid tissues, where it participates in the germinal center and maintains long-term infection primarily in memory B cells during the chronic phase of infection. Macrophage and B cells of the peritoneal cavity are also reservoirs of latency and reactivation. Reactivation cues lead to dissemination to mucosal surfaces for transmission to naïve animals. Viral factors that promote acute replication and dissemination in vivo are located in the left panel. Viral and host factors that promote latency and/or replication in vivo are located in the right panels. A summary of viral mutant phenotypes is provided in Supplemental Table 1, and detailed route-dependent and organ-specific phenotypes are provided in Supplemental Table 2. Abbreviations: ATG4, autophagy-regulating protease; ATM, ataxia-telangiectasia mutated; BAFFR, B cell activating factor receptor; Dnmt, DNA methyltransferase; H2AX, histone 2A family member X; HDAC, histone deacetylase; HIF, hypoxia-inducible factor; HOIL, hemoxidized iron-regulatory protein 2 ubiquitin ligase-1; IFN, interferon; IRF, interferon regulatory factor; LXR, liver X receptor; MHV68, murine gammaherpesvirus 68; miRNA, microRNA; mLANA, MHV68 latency-associated nuclear antigen; MyD88, myeloid differentiation factor 88; NF-κB, nuclear factor kappa B; ORF, open reading frame; RTA, replication and transcription activator; SHP1, Src homology region 2 domain-containing phosphatase-1; STAT3, signal transducer and activator of transcription 3; TLR, Toll-like receptor; TMER, transfer RNA-microRNA-encoded RNA; vGPCR, viral G protein–coupled receptor; vtRNA, viral tRNA. Figure adapted from images created with BioRender.com.

In vivo, MHV68 infection proceeds in discrete stages from the inoculation site to establishment of latency at numerous sites in the periphery, with return to mucosal tissues for transmission (Figure 3). In each stage, an array of virus gene products act in concert to usurp normal host biological processes and evade host immune responses.

### 2.1. Acute Phase of Infection

The natural route of MHV68 transmission remains unknown; however, owing to likely spread through respiratory and oral routes, most studies utilize intranasal (i.n.) inoculation. Inoculations under anesthesia result in highly reproducible infections in the lungs and subsequent spread to the periphery, while inoculation in the absence of anesthesia results in virus replication in the nasal cavity prior to spread (11). Additional studies use non-natural inoculation routes such as intraperitoneal (i.p.) to bypass mucosal barriers, allowing the assessment of infection determinants at peripheral sites without the restriction of dissemination bottlenecks. In parallel with i.n. inoculations, such studies allow important insight into the stage of infection at which viral factors act.

#### 2.1.1. Events in tissues of the host during acute infection.

At mucosal sites, MHV68 initially establishes a foothold in epithelial cells such as alveolar epithelial cells of the lungs (under anesthesia) or the olfactory epithelium of the nose (without anesthesia) (11). Macrophages play a cooperative role at the initial site of replication, with macrophage infection initiating upon internalization of virus particles presented by epithelial cells (12). Local infection results in productive replication in macrophage and then alveolar epithelial cells, with time to peak replication dependent upon inoculating dose; typical i.n. inoculations result in peak lung titers at 4–7 dpi, with virus largely undetectable by 10–12 dpi.

#### 2.1.2. Viral determinants of acute infection.

Many lytic gene products essential for robust replication in cell culture yield potent mutation phenotypes in vivo (reviewed in 13) (Supplemental Tables 1 and 2). For example, translational stop mutations in ORF50, which encodes the critical immediate early protein replication and transcription activator (RTA), result in replication-incompetent virus. Although infectious mutant virus can be grown on complementing cell lines, RTA-deficient viruses do not replicate in vivo (14). Similarly, ORF49 protein, a derepressor of RTA, is required for efficient lytic replication both in vitro and in vivo (15). Recent studies validated critical roles in the acute phase for lytic genes ORF35 (16), ORF48 (17), and ORF63 (18), in addition to genetic elements including the lytic origins of replication (oriLyt) (19).

Viral gene products essential for key facets of the acute phase, such as cell type–specific infectivity or immune evasion, cannot be accurately modeled in cell culture. For example, a virus carrying a mutation in the glycoprotein B furin cleavage site is marginally attenuated in fibroblasts in vitro but results in significantly reduced lytic infection in vivo due to a defect in alveolar macrophage infection (20). Although the viral uracil DNA deglycosylase (vUNG, ORF46) is not essential for replication in culture, a vUNG stop mutant is highly attenuated for acute replication in the lungs (21) and demonstrates a cooperative role with the viral dUTPase (22), supporting a model wherein uracil incorporation in viral genomes hinders productive replication in the more restrictive, nondividing cells of mucosal tissues. Finally, each of the gammaherpesviruses encodes at least one tegument protein with homology to a host purine synthesis enzyme; MHV68 encodes three, ORF75A, ORF75B, and ORF75C, which have distinct functions revealed through in vivo studies (23). ORF75C is best studied as it, like KSHV ORF75, disables host promyelocytic leukemia nuclear bodies (PML-NB) (24), in addition to its novel role in mediating the deamidation of retinoic acid-inducible gene I (RIG-I) to thwart inflammatory cytokine production (25).

### 2.2. Trafficking to Draining Lymph Nodes and Peripheral Dissemination

Following localized replication, MHV68 spreads to local draining lymph nodes (LN) primarily via dendritic cell transport (26). Following passage through dendritic cells, the virus infects other leukocytes in the draining LN, including B cells. This process requires some degree of lytic replication, as viruses deficient in essential lytic genes are unable to surmount mucosal barriers to traffic beyond regional sites. Although not yet rigorously demonstrated, it is presumed that B cells in the LN are latently infected in a manner consistent with latency establishment at peripheral sites.

#### 2.2.1. Events in tissues of the host during trafficking and dissemination.

The virus seeds peripheral sites through hematogenous dissemination. Rather than direct release of virus particles into circulation, efficient dissemination from mucosal sites requires entry of infected B cells into circulation (27). The ensuing entry of circulating, latently infected B cells, coupled with in situ reactivation (28,29), provides the primary means by which distal organs are seeded with infectious virus (reviewed in 13, 30).

Peripheral infection is commonly assessed in the spleen due to its rich source of B cells and ease of access; however, virus is also present in LNs, peritoneal cavity, liver, thymus, and bone marrow (31-34). The spleen is likely among the first organs infected due to its vascular filtration function. Following i.n. inoculation, virus remains undetectable until 7–8 dpi (28). Replication in myeloid cells precedes infection of B cells (12, 28). MHV68 also appears to pass through marginal zone B cells and/or follicular dendritic cells prior to reaching the follicular B cell compartment (12, 28). Following secondary amplification in peripheral organs, the acute phase of infection ends with infectious virus throughout the host reduced to a level below the limit of detection by 14–16 dpi.

#### 2.2.2. Viral determinants of trafficking and dissemination.

Recent studies have begun to elucidate the role of MHV68 genes in trafficking and dissemination. A striking example is the small ncRNA transfer RNA-microRNA-encoded RNA (TMER) 4. TMER4-deficient viruses undergo normal replication in the lung and traffic to the draining LN but demonstrate a significant reduction in infected cells in circulation (27) and an increase in infected cells in the draining LN (35). Remarkably, replacement of TMER4 with the EBV encoded small RNA (EBER) 1 fully restores virus fitness (35), implicating a conserved function of TMER4 and EBER1 as mediators of infected B cell egress and highlighting the draining LN as a critical bottleneck for hematogenous dissemination. Additional multifunctional virus proteins likely promote MHV68 dissemination. Cre-mediated deletion of virus genes in vivo reveals that MHV68 latency-associated nuclear antigen (mLANA) and M2 may facilitate GC B cell infection in draining LN, which in turn facilitates peripheral dissemination (36, 37).

### 2.3. Latency and Reactivation

Following dissemination, MHV68 establishes stable lifelong latency, a hallmark of the chronic phase of infection. The spleen is a major site, with up to 1 in 100 splenocytes harboring latent viral genome during the establishment phase (typically assessed at 16–18 dpi). The load of latently infected cells is independent of inoculation dose with doses ranging from 1 × 10^6^ to 0.1 PFU resulting in identical frequencies of latently infected cells.

#### 2.3.1. Events in host tissues that support latency and reactivation.

The primary cellular compartment for MHV68 latency in secondary lymphoid tissues is B cells. As for EBV, naïve follicular B cells are thought to be the initial target, with the virus driving infected cells through GC reactions into long-lived memory B cells (reviewed in 38) (Supplemental Figure 1). Consistent with this, during latency establishment, the virus genome is detectable in naïve follicular B2 B cells (sIgD^+^), GC B cells (GL7^+^/CD95^+^), and isotype-switched memory B cells (reviewed in 13, 39). Cumulative data indicate that MHV68-infected naïve B cells enter into the GC to undergo proliferative expansion prior to differentiation to class-switched memory B cells (see the sidebar titled Immunoglobulin Gene Repertoire). Reflecting this, the number of infected GC B cells rapidly expands from 13 to 18 days, and 60–80% of infected B cells carry GC markers during peak latency.

Within 4 weeks, coincident with cell-mediated immune control of infection, the frequency of latently infected cells begins to contract, achieving a stable level of approximately 1 in 10,000 by 6–8 weeks that is maintained for the life of the host. This maintenance phase of latency is defined by greatly diminished numbers of infected naïve B cells and stable maintenance of latency in the isotype-switched memory B cell compartment (40, 41). This identification of a latency setpoint is consistent with models of long-term EBV infection and strongly suggests that homeostatic mechanisms regulate the latency reservoir (reviewed in 42).

Although mature B2 B cells are the predominant reservoir for latency, B1 B cells and macrophages in the peritoneal cavity harbor latent virus during long-term infection (32, 43). In addition, stable infection of immature B cells in the bone marrow and transitional B cells in the spleen, each of which has a high turnover rate, suggests that recurrent infection of developing B cells contributes to homeostatic maintenance of the mature B cell compartment (34).

A key facet of herpesvirus latency is the ability to reactivate to the lytic cycle. MHV68 reactivation in vivo is not readily detectable. Instead, spontaneous reactivation from ex vivo samples can be monitored by limiting dilution reactivation or infectious center assays. Approximately 10% of infected splenocytes harvested during the establishment phase reactivate ex vivo (31, 44). During the maintenance phase few splenocytes reactivate, supporting the concept that transition to a tightly latent state occurs during latency contraction (44). Macrophage infection may represent a distinct form of latency, as this reservoir demonstrates greatly enhanced frequency of reactivation as late as 6 months pi (32). Like EBV and KSHV, MHV68 reactivation represents a key step in virus transmission. During sexual transmission between infected female and uninfected male mice, splenic reactivation precedes lytic replication in the vaginal epithelium (45). This transmission model provides a platform to examine the efficacy of antiviral interventions (46-48).

#### 2.3.2. Viral determinants of latency and reactivation.

Multiple MHV68 genes affect latent infection. Many of these genes, including ORF48 (17), ORF54 (dUTPase) (49), ORF63 (18), and ORF64 (50), promote lytic replication or virus trafficking and thus have compound downstream phenotypes during chronic infection. Here we focus instead on viral factors that have demonstrated clear effects on latency and/or reactivation, with special focus on those genes that are well conserved with EBV or KSHV. For a more comprehensive description of MHV68 genes that promote the chronic phase of infection, see Figures 1 and 3 and Supplemental Tables 1 and 2.

##### 2.3.2.1. MHV68 latency-associated nuclear antigen.

All gammaherpesviruses encode an episomal maintenance protein that is expressed during latency and is requisite for virus genome segregation in dividing cells. For MHV68, this protein is a latency-associated nuclear antigen (mLANA; ORF73), which shares homology with KSHV LANA (kLANA) and Epstein-Barr nuclear antigen 1 (EBNA1). Viruses deficient in mLANA display dramatic defects in latency and reactivation (13, 36). Studies using a *loxP*-flanked ORF73 recombinant virus enabled cell type–specific assessment of mLANA functions in B cells in vivo; the infection of mice expressing Cre under the activation-induced cytidine deaminase (AID) promoter revealed that mLANA expression in GC B cells is critical for latency establishment (36).

Structure and mutation studies have revealed the importance of conserved domains that contribute to mLANA function. The C-terminal region of mLANA carries a DNA binding domain reminiscent of EBNA1 (51) that interacts with the MHV68 terminal repeats to mediate episome persistence (52, 53). Mutation of this region significantly attenuates latency and reduces reactivation (51-53). mLANA also carries an E3 ligase domain essential for GC B cell expansion (54). Conservation between mLANA and kLANA facilitated studies examining precise domains of kLANA crucial for in vivo function (55-57) and revealed that kLANA and mLANA are functionally interchangeable for episome maintenance (58, 59).

##### 2.3.2.2. The latency determinant M2.

M2 protein plays a crucial role in mediating signaling pathways of infected B cells to facilitate latency and B cell differentiation. M2 exhibits functional overlap with EBV LMP1 and LMP2A and KSHV K1 despite its lack of homology. M2 acts as an adaptor to modify B cell signaling via interaction with SH2- and SH3-containing proteins. These interactions mediate numerous events, including activation of the nuclear factor of activated T cells (NFAT) pathway and interferon regulatory factor 4 (IRF4), key steps in the induction of IL-10 (60). IL-10 is an anti-inflammatory cytokine that also functions in MHV68 latency to promote B cell proliferation and differentiation (61) and to inhibit apoptosis (62). Consistent with the high level of M2 in GC B cells (61), specific deletion of M2 in AID-expressing GC B cells significantly impairs latency (37). MHV68 M2 mutants display a deficiency in infected plasma cells and reactivation in the spleen (63), and M2 expression alone promotes activated B cells to differentiate to GC B and plasma cells (61). M2 is not required following i.p. inoculation. Thus, the most essential functions of M2 may lie within GC reactions in the initial draining LNs, which seed the B cell reservoir to facilitate virus dissemination and reactivation at peripheral sites (37).

##### 2.3.2.3. V-cyclin.

Subversion of cell cycle inhibitors is a strategy shared among gammaherpesviruses. Like KSHV, MHV68 ORF72 encodes a v-cyclin homologous to cellular D-type cyclins that can interact with host cyclin-dependent kinases (CDKs) to promote cell cycle progression. KSHV v-cyclin substitutes for MHV68 v-cyclin in vivo, indicating functional conservation (64). V-cyclin plays a key role in the ability of latently infected cells to undergo reactivation in part due to its ability to antagonize the host CDK inhibitor p18^INK4c^ (65). Knocking out or attenuating p18^INK4c^ restores the reactivation defect of v-cyclin knockout virus (65) and replacement of v-cyclin with host p18^INK4c^ phenocopies defects of v-cyclin mutant virus in reactivation and pneumonia (66).

##### 2.3.2.4. vBcl-2.

Each gammaherpesvirus encodes at least one homolog of the cellular anti-apoptotic protein Bcl-2. The MHV68 vBcl-2 protein (M11) is a functional inhibitor of both apoptosis and autophagy. MHV68 carrying vBcl-2 mutations functions normally during acute replication but displays moderate defects in latency and reactivation due to these dual functions (reviewed in 13). vBcl-2 may be particularly important for survival of infected immature B cells in the bone marrow and transitional B cells in the spleen, which normally display low levels of host Bcl-2 as they undergo selection (34). Notably, in vivo depletion of developing B cells significantly reduces mature B cell latency, suggesting that homeostatic maintenance of the latency reservoir is at least partially sustained through recurrent infection and vBcl-2-mediated survival of developing B cells (34).

#### 2.3.3. Host determinants of latency and reactivation.

A growing list of host factors have been found to promote or antagonize MHV68 latency and reactivation. For example, the tyrosine phosphatase SHP1 attenuates B cell receptor signaling yet its loss in B cells leads to a decrease in MHV68 latency that coincides with a loss of germinal center expansion (67). In contrast, nuclear liver X receptor alpha counteracts MHV68 infection by altering MHV68 replication and myeloid cell tropism (68, 69). Here we summarize insights into major pathways and host factors that the virus requires to establish a foothold in long-term latency reservoirs.

##### 2.3.3.1. Nuclear factor kappa B.

The nuclear factor kappa B (NF-κB) signaling pathway drives interferon (IFN) and inflammatory cytokine production, in addition to playing a role in the activation, proliferation, and differentiation of B cells. Targeting the canonical NF-κB signaling pathway dramatically reduces latency in B cells, implicating NF-κB activation as a central step in latent infection (reviewed in 70). Nevertheless, NF-κB signaling is likely downregulated during specific phases of infection, as both mLANA and MHV-68 RTA target NF-κB subunit RelA/p65 for degradation. Mutation of the mLANA E3L domain abrogates MHV68-driven GC B cell infection, implicating mLANA E3L activity in GC B cell proliferative expansion (54). Toll-like receptor (TLR)-induced NF-κB activation disrupts an established latency program to increase genome-positive cells or reactivation in vivo (70). Consistent with this, RTA binding to the oriLyt is enhanced upon lipopolysaccharide stimulation (71).

##### 2.3.3.2. Signal transducer and activator of transcription 3.

Mice lacking signal transducer and activator of transcription 3 (STAT3) in CD19^+^ B cells demonstrate dramatically reduced MHV68 latency (72). This defect remains throughout the maintenance phase of infection (72), which is reminiscent of the pronounced phenotype in CD40-deficient mice (73). STAT3 is induced in response to IFNs, as well as key cytokines such as IL-6, IL-10, and IL-21. MHV68 activates STAT3 via M2-induced cellular IL-10 (74). Further, IL-21 produced by T follicular cells in MHV68-infected animals promotes GC expansion and MHV68 latency (75). Similarly, IL-16 is upregulated in the serum of infected mice and inhibits reactivation through the STAT3-p21 axis (76). Direct gene targets of STAT3 are not known in the context of MHV68 infection, but the interaction of STAT3 with the lytic transactivator RTA is enhanced by IL-6 (77).

##### 2.3.3.3. Plasma cell factors.

Plasma cells represent a major source of reactivating virus, in large part because, as is the case for EBV and KSHV, transcription factors expressed in plasma cells are triggers for reactivation (63). Consistent with this, Blimp1 and IRF4, major regulators of plasma cell differentiation, are required for splenic reactivation (78,79). In contrast, X-box binding protein 1, a plasma cell factor that induces the expression of lytic transactivators of gammaherpesviruses, is surprisingly dispensable for MHV68 reactivation in vivo (79). Host microRNA-155 plays a B cell–intrinsic role critical for reactivation (80), perhaps due to roles in plasmablast proliferation and survival.

##### 2.3.3.4. Hypoxia inducible factor 1 alpha.

Hypoxia inducible factor 1 alpha (HIF1α) acts as an oxygen sensor to upregulate genes that facilitate cell survival and anerobic metabolism in oxygen-deprived tissues. B cells transiting through GCs are exposed to hypoxic conditions, and KSHV lesions are notable for their presentation in lower extremities where low oxygen predominates. HIF1α transactivates gammaherpesvirus lytic switch proteins, and ex vivo reactivation of MHV68 from splenocytes is accelerated in hypoxia (81). In addition, conditional HIF1α deletion in vivo decreases lytic replication in the lungs and reactivation from the spleen (81).

##### 2.3.3.5. Ataxia-telangiectasia mutated.

Host ataxia-telangiectasia mutated (ATM) protein kinase is critical for responding to double-stranded DNA breaks and has roles in inflammation and virus control. While MHV68 infection induces an ATM-dependent p53 response at an early stage of lytic infection in culture, downstream p53 target genes and exogenous triggers of apoptosis are blocked by mLANA at later times, which protects the infected cells from p53-mediated cell death (9). Depletion of ATM in B cells in vivo leads to decreased MHV68 latency likely through impairing the differentiation of ATM-deficient B cells (82). Myeloid cells deficient in ATM lead to reduced reactivation of MHV68 upon explant through an unknown mechanism (83).

## 3. IMMUNE CONTROL OF MHV68 INFECTION

MHV68 infection is an ideal platform to study immune control of gammaherpesvirus infections (reviewed in 13, 30, 84). Studies reveal immune factors that regulate chronic infection and evasive strategies used by these viruses to block immune control. This insight is harnessed for new vaccination approaches.

### 3.1. Innate Immune Control

Type I IFN cytokines dampen both acute replication and reactivation from latency; their potency is evident in the mortality that manifests in MHV68-infected mice lacking IFNαβ receptor or STAT1 (85). Type III IFN suppresses MHV68 replication in the olfactory epithelium (86). Host factors that sense viruses and drive IFN-mediated defense, including GMP-AMP synthase (cGAS), TLR7, TLR9, IRF1 (87), IRF3, and IRF7, restrict lytic replication in vitro and in vivo (reviewed in 88). Innate responses also regulate chronic infection. IRF7 restricts latency in peritoneal B cells (89), and mice lacking both TLR7 and TLR9 exhibit increased reactivation and viral load from splenocytes (90).

Host factors involved in IFN signaling inflammation also have proviral roles. B cell–specific depletion of IRF1 decreases virus colonization of the spleen that coincides with reduced GC responses and virus-specific antibodies (91). IFN-induced transmembrane protein 1 promotes acute replication of MHV68 in the lungs, consistent with proviral roles uncovered for EBV and KSHV in virus entry (92). Mice lacking the IL-1R exhibit defects in levels of MHV68 latency, in addition to a decreased GC response and polyclonal antibody response (93).

Cells of the innate immune system coordinate with the adaptive immune system to control acute replication and limit virus reactivation from latency. Conventional dendritic cells prime Th responses in the draining LN after intranasal infection (94), while macrophages contribute to the inflammatory response that recruits immune cells and has direct effect on infected cells via IFNγ and tumor necrosis factor-α. Inflammatory responses may hinder IFN responses: Reactive oxygen species oxidize stimulator of interferon genes (STING) to block its polymerization and downstream IFN induction, leading to enhanced lytic MHV68 replication in macrophages (95).

MHV68 encodes numerous modulators of IFN responses including tegument protein ORF11, protein kinase ORF36, ORF54, and the M2 latency protein. In addition, the ORF64 tegument protein stabilizes incoming capsids and impairs cGAS-STING sensing of virion DNA. Loss of ORF64 deubiquitinase activity leads to an increase in IL-1β and type I IFN production, and this mutant has a latency defect that is restored in STING^−/−^ mice (50). Investigations in mice lacking inflammasome components Caspase1 and Caspase11 (96) and the cytidine deaminase apolipoprotein B editing complex 3 (APOBEC3) (97, 98) did not reveal antiviral roles against wild-type MHV68; their roles may not be apparent if viral modulators counteract their restrictive action.

### 3.2. Cell-Mediated Immune Control

CD4 T cells play an essential role in regulating long-term gammaherpesvirus infections (reviewed in 13, 30), as indicated by the high incidence of gammaherpesvirus malignancies in human acquired immunodeficiency syndrome patients with CD4 T cell loss. MHV68 infection of mice lacking CD4 T cells leads to uncontrolled MHV68 reactivation in mice and eventual death. CD4 T cells directly regulate MHV68 infection through release of IFNγ, direct cytotoxic functions (99), and suppression of myeloid cell infection (100) (Supplemental Figure 2). In addition, CD4 T cells play a critical role in regulating long-term infection by providing CD8 T cell help, in part via CD40L activation of antigen-presenting cells (84). CD4 T cells also promote generation of virus-specific antibodies and natural killer cell infiltration to control virus replication in the lungs (101). During acute lung infection, CD4 T cells serve as a major source of immunosuppressive IL-10, leading to reduced infiltration of myeloid cells and activated CD4 T cells (102). IL-21 is produced by CD4 T follicular helper cells that drive GC reactions. IL-21R on the infected B cells is critical for latency amplification in the spleen (75).

CD8 T cells primarily exert control through direct targeting of latently infected cells (reviewed in 13, 30). Although CD8 T cells participate in the acute phase to restrict infection, lytic replication is fully controlled even in their absence. CD8 T cell effector mechanisms, including perforin, granzyme, and FasL, regulate the number of infected cells during acute infection and latency (Supplemental Figure 2). IFNγ produced by CD4 and CD8 T cells (as well as myeloid cells) is essential for regulating reactivation through IFNγ/STAT1-responsive elements in the RTA promoter (reviewed in 13). Notably, loss of autophagy genes in the myeloid compartment heightens inflammation and IFNγ production, and it suppresses MHV68 reactivation (103).

CD8 T cells respond to multiple MHV68 epitopes, including at least two waves of responses to lytic protein epitopes and a response to the M2 latency protein (104). CD8 T cells control lymphoma cells expressing viral epitopes in MHV68-infected mice (105, 106). Antiviral CD8 T cells do not display functional exhaustion in immunocompetent mice (84). Interestingly though, PD-1 is upregulated on antiviral CD8 T cells in mice that lack CD4 T cells, leading to an altered hierarchy wherein CD8 T cells that respond to subdominant epitopes provide compensatory control (107). While inhibitory markers of exhaustion are upregulated on CD8 T cells in the absence of CD4 T cells, their expression on CD8 T cells during acute infection in normal mice suggests more studies are required to differentiate activation from exhaustion (84).

B cells and antibody contribute to control of MHV68 infection: B cell–deficient mice demonstrate increased reactivation from non-B cell reservoirs of latency as well as ongoing persistent virus replication (44). Conversely, transfer of antiviral antibody decreases the number of latently infected cells and inhibits reactivation (reviewed in 13, 30). Virus-specific antibodies block secondary infection of the olfactory epithelium; neutralizing antibodies are not sufficient to block infection or control virus reactivation from the vaginal mucosa, indicating site-specific functions (108). MHV68 colonization of the spleen drives polyclonal B cell activation and a transient burst of self-reactive antibodies (109) that together impede the antiviral antibody response. B cells isolated from MHV68-infected mice are suppressed for antibody responses, in part due to virus-mediated upregulation of PTEN, which suppresses BCR-mediated activation of the PI3K pathway (110).

MHV68 is used in genetically modified mice to model infection and host responses that closely mimic aspects of human immune deficiencies and immune therapies (13, 111, 112). For example, humans with an autosomal dominant mutant form of human STAT1 (R274W) have heightened susceptibility to herpesvirus infections, and MHV68 infection of mice heterozygous for STAT1 R274W recapitulates this phenotype. CD4 and CD8 T cell responses are impaired in these animals, yet virus is controlled in mixed bone marrow chimeric mice when wild-type leukocytes are present. Thus, defective antigen-specific CD8 T cell responses likely contribute to the R274W immunodeficiency observed in humans (113).

### 3.3. Vaccines to Control Gammaherpesvirus Infection and Disease

The murine system enables investigators to capitalize on the knowledge of MHV68 genes, most with direct counterparts to KSHV and EBV, to test rational preventive or therapeutic vaccine designs that provide sterilizing immunity or control replication and reactivation, respectively. Reactivation-defective viruses protect against wild-type virus challenge (114) more effectively than vaccine strategies geared toward specific glycoproteins or latent epitopes (reviewed in 30). Recent studies refined this approach to generate live-attenuated vaccines defective for viral latency. In one case, an MHV68 recombinant lacking ORF73 and genes of the MHV68 unique latency locus was generated (115). In another, a complex recombinant was constructed carrying stop insertions in genes with dual roles in lytic replication and immune evasion (ORF10, ORF36, ORF54, mK3), along with replacement of the MHV68 conserved latency locus (ORF72, M11, ORF73, ORF74) with a constitutive RTA expression construct (116). Both designs provide protection from wild-type virus challenge through induction of synergistic CD4 T cell, CD8 T cell, and antibody responses (115, 116). Rational design of attenuated MHV68 viruses shows promise in generating full-spectrum protective immune responses against gammaherpesvirus infections.

## 4. PATHOGENESIS

### 4.1. Oncogenesis Models

**Figure 4:**
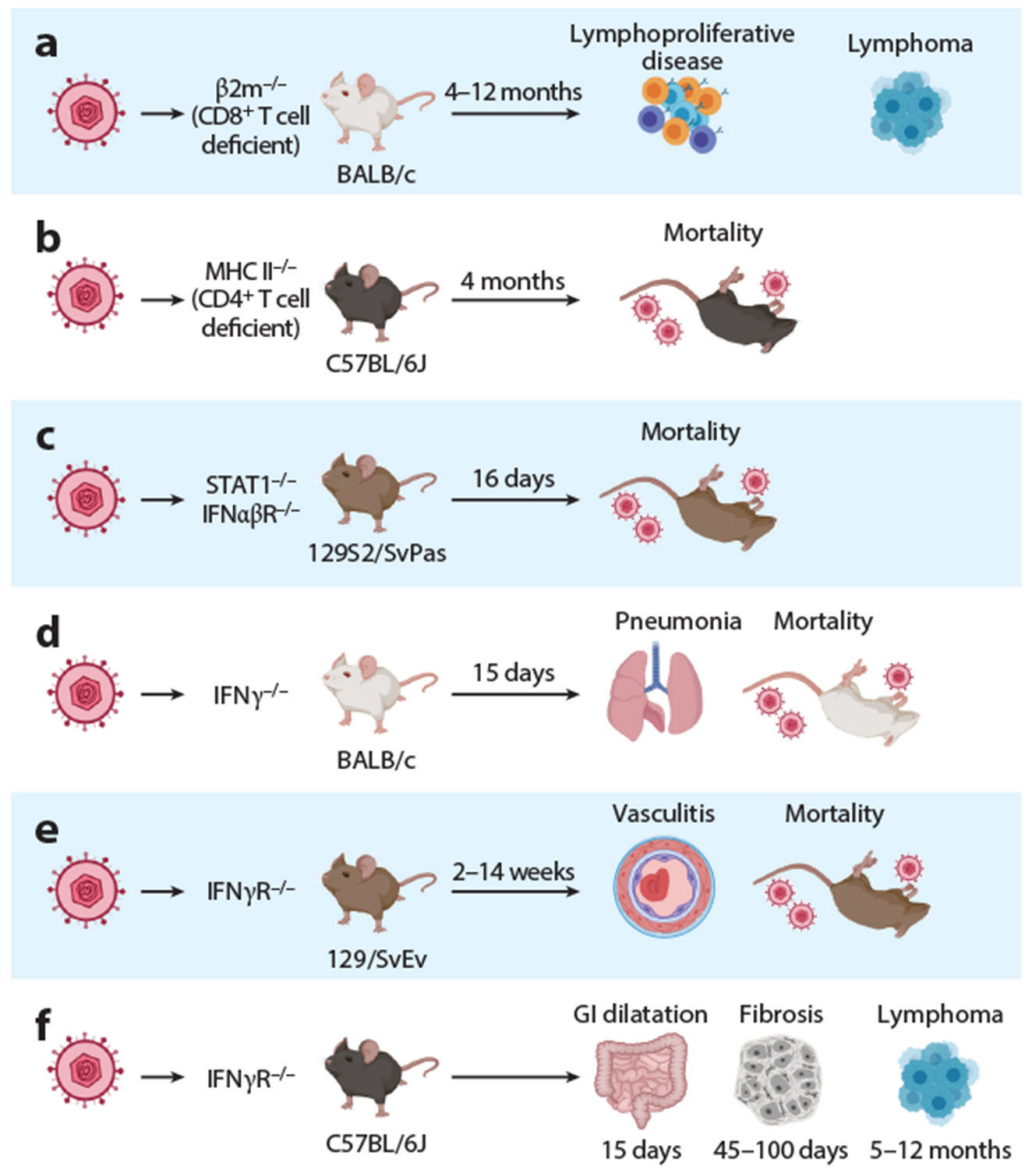
MHV68 infection of immune-compromised mice leading to disease and mortality. (*a*) Lymphoproliferative disease precedes the development of lymphomas in β2m^−/−^ mice that are largely deficient in CD8^+^ T cells. MHV68 v-cyclin and vBcl-2 promote disease (118, 120). (*b*) MHC II^−/−^ mice that are CD4^+^ T cell deficient succumb within 4 months of infection (123). (*c*) Infection of IFNαβR^−/−^ or STAT1^−/−^ mice defective in type I IFN signaling leads to a rapid onset of mortality (85). (*d*) Infected BALB/c mice that fail to produce IFNγ develop lethal pneumonia by 15 dpi in a v-cyclin-dependent manner (124). (*e*) The infection of IFNγR^−/−^ mice on a 129/SvEv background leads to a slower progression of large vessel vasculitis that leads to mortality over a 2- to 14-week period (128). (*f*) The infection of IFNγR^−/−^ mice on a C57BL/6J background leads to a range of pathologies including GI dilatation (127), fibrosis (126), and lymphoma (119). Abbreviations: GI, gastrointestinal; IFN, interferon; MHC II, major histocompatibility complex class II; MHV68, murine gammaherpesvirus 68; STAT1, signal transducer and activator of transcription 1. Figure adapted from images created with BioRender.com.

As for EBV and KSHV, inoculation of fully immunocompetent hosts with MHV68 typically results in asymptomatic infection. For example, although EBV infects greater than 90% of the human population, only a small frequency of those infected develop EBV-associated malignancies. However, as evidenced by the vastly increased prevalence of EBV- and KSHV-associated tumors in human immunodeficiency virus (HIV)–infected individuals and transplant recipients, disruption of key host immune factors leads to a variety of deleterious outcomes that favor the virus (Figure 4). Likewise, while B cell lymphomas arise at a penetrance of 9% in wild-type mice inoculated with MHV68 over 3 years, the frequency of lymphoma in wild-type mice treated with immunosuppressive agents is markedly higher (117). Consistent with the role of CD8 T cells in regulating EBV tumors, infection of CD8 T cell–deficient (β2 microglobulin^−/−^, β2m^−/−^) BALB/c mice reproducibly results in B cell lymphoproliferative disease or B cell lymphoma with a cumulative penetrance above 80% by 12–14 months (118). Early-stage lymphoproliferative lesions are polyclonal, while late-stage lymphomas appear monoclonal and display features reminiscent of large B cell lymphomas (118). Interestingly, MHV68 infection of IFNγ receptor–deficient (IFNγR^−/−^) mice results in pulmonary B cell lymphoma (119).

A powerful aspect of MHV68 tumor models is the ability to examine the contribution of virus genes to tumorigenesis. For example, MHV68 v-cyclin and vBcl-2 contribute to enhanced disease in β2m^−/−^ BALB/c mice, consistent with their roles as negative regulators of virus reactivation (120). In keeping with the function of the KSHV viral G protein–coupled receptor (vGPCR) as a more potent activator of downstream signaling than the MHV68 vGPCR, immunosuppressed mice inoculated with recombinant MHV68 carrying KSHV vGPCR in place of MHV68 vGPCR develop angiogenic lesions that resemble Kaposi sarcoma (121). MHV68 infection drives immortalization of primary murine fetal liver B cells, and mLANA and v-cyclin both contribute to this process (122). Notably, transplant of MHV68 immortalized cells to athymic nude or Rag2-deficient mice results in metastatic lymphomas that resemble KSHV B cell lymphomas (122).

### 4.2. Other Disease Models

In contrast to the asymptomatic infection observed in immunocompetent mice, infection of mice lacking immune factors drives pathologies that lead to morbidities that recapitulate aspects of human disease (reviewed in 111) (Supplemental Figure 2). Rapid death is observed upon infection of mice that lack type I IFN receptors or IFN-responsive STAT1 due to uncontrolled virus replication (85). A slower progression to death occurs in CD4 T cell–deficient mice that are unable to control persistent infection (123). The loss of chronic virus control in MHV68-infected mice lacking IFNγ signaling leads to an array of pathologies, dependent on background genetics. IFNγ^−/−^ mice on a BALB/c background develop lethal pneumonia when infected with wild-type MHV68 but not mutants lacking v-cyclin, vBcl-2 (124), or the TMER-derived microRNAs (125). IFNγR deficiency on the C57BL/6J background leads to multiorgan fibrosis (126), lymphoproliferation and frank lymphoma (119), and gastrointestinal dilatation (127). IFNγR deficiency on the 129Sv/Ev background induces large vessel vasculitis (128). Fibrosis of the lungs is also modeled by MHV68 infection following bleomycin instillation of BALB/c mice or MHV68 infection of aged C57BL/6J mice (reviewed in 129). Examination of viral mutants and host factors has revealed key virus-host interactions that promote fibrosis, vasculitis, pneumonia, and arthritis (111, 130, 131). Interestingly, the gut microbiome differentially influences MHV68-driven pathologies (132, 133). Such models serve as a foundation to explore therapeutics that impair virus-driven inflammatory processes in the specific tissues that relate to human disease (127, 132).

### 4.3. Coinfection Models

Single pathogen infections fall short of modeling the complexity of the host biome. The outcome of coinfection of MHV68 with other pathogens varies with the stage and order of infection (reviewed in 134) (Supplemental Figure 3). Latent MHV68 infection induces an inflammatory milieu that is protective against numerous bacterial pathogens (135, 136) and virus infections (13, 137). In many cases, this protection is mediated by elevated IFNγ or other immune factors induced by chronic MHV68 infection. However, infection with MHV68 followed by influenza and helminth challenge reduces vaccine-induced antibody responses (138).

KSHV and EBV cancers are leading causes of mortality where malaria coinfection rates are high. Notably, the nonlethal mouse malarial pathogen *Plasmodium yoelii* XNL leads to 100% lethality in mice undergoing acute MHV68 infection (139). Dually infected animals are suppressed for GC and T follicular helper responses that correlate with a reduction in parasite-specific antibodies, an effect that is dependent upon MHV68 latency protein M2 (139).

Coinfecting pathogens may likewise alter the course of MHV68 infection. Helminth infection after established MHV68 infection induces IL-4 that promotes MHV68 reactivation (140). In contrast, helminth infection prior to MHV68 infection decreases acute virus replication due to enhanced antiviral CD8 T cell responses (141). Multi-infection approaches reveal host mechanisms that modulate herpesvirus latency (134) and imply that vaccine efficacy might be more rigorously tested during coinfections (138).

## 5. CONCLUDING REMARKS

MHV68 infection of mice offers a robust and highly tractable system to examine all aspects of in vivo gammaherpesvirus infections, ranging from the complex molecular details of virus-driven B cell differentiation to the multifaceted interplay between virus and host factors during chronic infection. As evidenced by new findings demonstrating conservation of gene function and pathogenic strategies, it is clear that lymphotropic gammaherpesviruses endemic to rodent populations have much to teach us regarding KSHV and EBV pathogenesis. Future research using the MHV68 system will continue to provide a deeper understanding of the specific virus and host mechanisms that govern in vivo gammaherpesvirus infections and disease. MHV68 serves as a powerful model for preclinical tests of experimental antivirals, lytic induction strategies, immunotherapies, and vaccines.

## Supplementary Material

Supp Table 2

Supp Table 1

Supp Figs 1-3

## MHV68:

murine gammaherpesvirus 68, common isolate, referred to as *γ*HV68 or MuHV-4; formally annotated as murid gammaherpesvirus 4, MuGHV-4

## Latency:

state of infection where viral genome is maintained as an episome with minimal viral gene expression and no infectious particle production

## Pathogenesis:

strictly defined as the process by which the virus drives pathology; loosely applied as the process of host colonization

## Acute phase of infection:

typically defined as 1- to 12-day period of active replication after inoculation of mucosal site

## TMER:

MHV68 genes that encode a transfer RNA-like molecule fused to one or more microRNA stem loops, transcribed by RNA polymerase III

## Chronic phase of infection:

loosely applied to describe a period wherein latency is established, although reactivation may occur concomitantly

## Limiting dilution assay:

sensitive, PCR-based assay to enumerate frequency of intact cells that harbor viral genome

## Reactivation:

switch from latent to productive infection; may occur spontaneously in host or upon explant; may be induced

## Infectious center assay:

plaque-based assay that enumerates frequency of infectious particles derived from tissues, measures preformed, and reactivated virus

## NONCODING RNAS

Murine gammaherpesvirus 68 (MHV68) encodes a wide breadth of noncoding RNAs (ncRNAs), ranging from 10-kb long ncRNAs (lncRNAs) to 21-nt microRNAs (miRNAs) (Figure 2). As evidenced by the conserved activity of MHV68 transfer RNA-miRNA-encoded RNA (TMER) 4 and Epstein-Barr virus (EBV) encoded small RNA (EBER) 1, many of these molecules likely exhibit functional overlap with EBV and Kaposi sarcoma herpesvirus (KSHV) ncRNAs. Consistent with this, of 1,505 identified host messenger RNA targets of MHV68 miRNAs, 86% are shared with EBV and/or KSHV (142). And a circular RNA (circRNA) identified in MHV68 exhibits features of EBV and KSHV circRNAs (143). Recent studies have begun to elucidate in vivo functions of MHV68 ncRNAs: MHV68 miRNAs contribute to establishment of memory B cell latency (125) and may alter long-term latency (144). The miRNA *mghv-miR-7–5p* suppresses host Ewing sarcoma breakpoint region 1 in vivo to promote germinal center B cell latency (145). TMER molecules, including a processed viral tRNA element itself, contribute to pneumonia pathogenesis (27, 125, 146). To add to the complexity, miRNA regulation of a viral lncRNA regulates the acute phase of infection (147). Future research will undoubtedly continue to unveil surprising functions for ncRNAs in gammaherpesvirus pathobiology.

## IMMUNOGLOBULIN GENE REPERTOIRE

High-throughput sequencing of the immunoglobulin genes indicates that infected B cells participate in the germinal center (GC), with evidence of somatic hypermutation and isotype class switching (38, 148, 149). There is a striking occurrence of clonal expansion and recurrence of Ighv10-1 usage in infected GC B cells, with little clonal overlap of V genes in uninfected B cells of the same animal at the peak of splenic latency. Infected B cells have a skew toward lambda light chain usage, as reported for tonsillar B cells newly infected with Kaposi sarcoma herpesvirus (150). The origin of plasmablasts and the link between GC reactions and long-term latency in memory B cells are unknown. The potential for the selection and expansion of virus nonreactive and autoreactive pathological B cells is an important issue for the field (reviewed in 38).

## SUMMARY POINTS

1. Gammaherpesvirus infection in the host is dynamic and complex, involving trafficking between mucosal sites and peripheral lymphoid tissues. Multiple rounds of lytic replication, latency, and reactivation transpire in cells of the hematopoietic and stromal compartments before latency is established in the memory B cell compartment.
2. Engagement of the germinal center (GC) is a key aspect of establishing latency, but the role of GC-experienced infected B cells in the long-term maintenance of latency is less understood.
3. Viral noncoding RNAs are a diverse family of molecules that directly participate in conserved aspects of gammaherpesvirus biology.
4. Host factors that control proliferation, cell survival, and differentiation of B cells can be targeted to reduce viral load and prevent gammaherpesvirus disease.
5. During coinfection, gammaherpesviruses and other pathogens influence the pathobiology of one another by altering effector functions of immune cells and by regulating viral gene expression via changes in the cytokine milieu.

## FUTURE ISSUES

1. The discovery of alternate transcript structures has uncovered an unprecedented layer of coding and regulatory potential in gammaherpesviruses that will be leveraged to mechanistically link extracellular signaling cues to controls of latency and reactivation.
2. Technological advancements including mass cytometry and single-cell sequencing will provide higher resolution of gammaherpesvirus latency reservoirs, better define the lineage of effector cell subsets, and define how the virus reprograms the cell to support latency.
3. Powerful genetic approaches including conditional cell-specific recombination and CRISPR strategies will inform the spatial and temporal roles of virus and host genes.
4. The generation of chimeric viruses wherein an Epstein-Barr virus or Kaposi sarcoma herpesvirus gene replaces the murine gammaherpesvirus 68 (MHV68) homolog will continue to inform studies of the human gammaherpesvirus counterparts.
5. The study of individual virus genes will advance the development of refined vaccine platforms that provide protection through induction of broad humoral and cell-mediated immunity.
6. Deeper study using MHV68-induced tumor models will reveal critical determinants of gammaherpesvirus oncogenesis.
