# Supplementary material for: Conquering the Host: Determinants of Pathogenesis Learned from Murine Gammaherpesvirus 68
Source: Annu Rev Virol. Author manuscript; Available in PMC 2022 May 31. (PMC9153731; doi:10.1146/annurev-virology-011921-082615)
Supplement: Supp Table 1 [file NIHMS1807025-supplement-Supp_Table_1.pdf]

Supplemental Table 1. Summarized role of MHV68 genes in promoting replication in culture and chronic infection in mice. [a]

Gene name	General function [b]	Kinetic class in lytic replication [c]	Latent expression [d]	Mutant virus description [e]	Lytic replication in vitro [f, g]	In vivo phenotypes of mutant viruses [h]	References
**All TMERs**	Noncoding RNA	Constitutive RNA Pol III-driven	yes	TMER.TKO mutant lacking all eight TMERs	wt	Decreased pneumonia in IFNγ$^{-/-}$ mice	(1)
				sncRNA.KO mutant lacking all vtRNAs and miRNAs	wt	Increased splenic latency in the maintenance phase after i.n.	(2)
**All miRNAs**	Noncoding RNA	Constitutive RNA Pol III-driven	yes	Zt6 mutant lacking all 14 pre-miRNA stem-loops	wt	Decreased splenic latency establishment and reactivation after i.n; decreased pneumonia in IFNγ$^{-/-}$ mice	(3)
**TMER1 (vtRNA1, miR-1, and miR-10)**	Noncoding RNA	Constitutive RNA Pol III-driven	yes	TMER1-only mutant expressing TMER1 but lacking all other seven TMERs	nd	Increased pneumonia in IFNγ$^{-/-}$ mice	(1)
				vtRNA1-only mutant expressing vtRNA1 but lacking miR-1 and miR-10 pre-miRNA stem-loops and all other seven TMERs	nd	Increased pneumonia in IFNγ$^{-/-}$ mice	(1)
**TMER2 (vtRNA2, miR-2, and miR-3)**	Noncoding RNA	Constitutive RNA Pol III-driven	yes	ΔmiR2 mutant lacking miR-2 pre-miRNA stem-loop	nd	wt	(4)
				ΔmiR2.3 mutant lacking both miR-2 and miR-3 pre-miRNA stem-loops	nd	wt	(4)
**TMER3 (vtRNA3 and miR-4)**	Noncoding RNA	Constitutive RNA Pol III-driven	yes	nd	nd	nd	n/a
**TMER4 (vtRNA4, miR-5, and miR-6)**	Noncoding RNA	Constitutive RNA Pol III-driven	yes	ΔmiR5.6 mutant lacking both miR-5 and miR-6 pre-miRNA stem-loops	wt	Severely decreased hematogenous dissemination and downstream latency establishment in the spleen and	(4)

Supplemental Material: Annu. Rev. Virol. 2021. 8:349-371
https://doi.org/10.1146/annurev-virology-011921-082615
Conquering the Host: Determinants of Pathogenesis Learned from Murine Gammaherpesvirus 68
Wang, Tibbetts, and Krug

						PECs after i.n.; decreased pneumonia in IFNγ$^{-/-}$ mice	
**TMER5 (vtRNA5, miR-7, and miR-12)**	Noncoding RNA	Constitutive RNA Pol III-driven	yes	ΔmiR7.12 mutant lacking both miR-7 and miR-12 pre-miRNA stem-loops	wt	Decreased splenic latency establishment after i.n.	(5)
				ΔmiR7 mutant lacking miR-7 pre-miRNA stem-loop	wt	Decreased splenic latency establishment after i.n.	(5)
				ΔmiR12 mutant lacking miR-12 pre-miRNA stem-loop	wt	wt	(5)
**TMER6 (vtRNA6, miR-13, and miR-8)**	Noncoding RNA	Constitutive RNA Pol III-driven	yes	nd	nd	nd	n/a
**TMER7 (vtRNA7 and miR-14)**	Noncoding RNA	Constitutive RNA Pol III-driven	yes	nd	nd	nd	n/a
**TMER8 (vtRNA8, miR-15, and miR-9)**	Noncoding RNA	Constitutive RNA Pol III-driven	yes	ΔmiR15.9 mutant lacking both miR-15 and miR-9 pre-miRNA stem-loops	wt	Increased lytic replication in the lungs after i.n.	(4, 6)
				ΔmiR9 mutant lacking miR-9 pre-miRNA stem-loop	nd	wt	(4)
**M1**	Secreted superantigen-like protein	Early-late	yes	M1.LacZ mutant deficient in the expression of M1 protein	wt	Increased reactivation in the spleen and PECs after i.p.; decreased lethality and fibrosis in IFNγR$^{-/-}$ mice	(7)
				M1.Δ511mutant deficient in the expression of M1 protein	nd	Decreased reactivation in the spleen after i.p.; increased reactivation in the PECs after i.p.; increased latency establishment in IgD- B cells after i.p.; increased LPD in β2m$^{-/-}$ mice	(7–10)

				M1.stop mutant deficient in the expression of M1 protein	nd	Decreased reactivation in the spleen after i.p.; increased reactivation in the PECs after i.p.	(8, 10)
**M2**	Adaptor protein	Early-late	yes	M2.LacZ mutant deficient in the expression of M2 protein	wt	Decreased splenic latency establishment and reactivation after i.n.; Increased reactivation in the PECs after i.p.	(11)
				M2.stop mutant deficient in the expression of M2 protein	nd	Decreased splenic latency establishment and reactivation after i.n. and i.p.	(11–13)
				76(RΔM2) mutant deficient in the expression of M2 protein	wt	Decreased splenic latency and/or reactivation after i.n.	(14)
				M2.FS mutant deficient in the expression of M2 protein	wt		(15)
				M2.loxP mutant carrying two loxP sites flanking M2 that enables conditional deletion of M2 [i]	wt	Decreased splenic latency establishment and reactivation after i.n in CD19-Cre mice; decreased splenic reactivation after i.p. in CD19-Cre mice	(13)
				M2.P8, M2.P9, and M2.P8/9 mutants harboring mutations in P8 and/or P9 PXXP motifs	nd	Decreased splenic latency establishment and reactivation after i.n.	(16)
				M2.Y120/129D mutant carrying aspartic acid substitutions for tyrosines 120 and 129 of M2	nd		(16)

				M2.Y129F/P7 mutant carrying phenylalanine substitution for tyrosine 129 of M2 and alanine substitutions for prolines 160 and 163 in the P7 motif	nd		(16)
**M3**	Chemokine binding protein	Early-late	yes	M3.stop mutant deficient in the expression of M3 protein	wt	Severely decreased lethality in CD1 mice	(17)
				M3.stop mutant deficient in the expression of M3 protein [j]	nd	Decreased splenic latency establishment and maintenance in wood mice after i.n.	(18)
**M4**	Secreted protein	Immediate early	yes	M4.stop mutant deficient in the expression of M4 protein	wt	Decreased splenic latency establishment and reactivation after i.n. and i.p.	(19)
**ORF4**	Glycoprotein (gp70), regulator of complement activation	Early-late	yes	ORF4.stop mutant deficient in the expression of ORF4 protein	wt	Decreased lethality in CD1, IFNγ$R^{-/-}$, F1, and Factor $B^{-/-}$ mice	(20)
				ORF4 deletion mutant	↓~10x	Decreased lytic replication in the lungs and downstream splenic latency and/or reactivation after i.n.	(21, 22)
**ORF6**	Single-stranded DNA binding protein (ssDNABP)	Early/Early-late	no	ΔssDNABP mutant deficient in the expression of ORF6 protein	essential	Severely decreased latency establishment in the spleen after i.n. and i.p.; severely decreased reactivation in the PECs after i.p.; severely decreased lethality in $Rag1^{-/-}$ mice	(23)
**ORF7**	DNA packaging terminase subunit 2	Early-late	no	nd	nd	nd	n/a

**ORF8**	Envelope glycoprotein B (gB)	Late	no	ORF8.ΔFCS mutant carrying deletion and/or mutations in the furin cleavage site that is deficient in gB cleavage activity	wt	Decreased lytic replication in the lungs after i.n.	(24)
**ORF9**	DNA polymerase catalytic subunit	Early/Early-late	no	nd	nd	nd	n/a
**ORF10**	Protein G10	Early-late	no	nd	nd	nd	n/a
**ORF11**	Virion protein G11/p43	Late	no	ORF11.stop mutant deficient in the expression of ORF11 protein	wt	Decreased lytic replication in the lungs after i.n.	(25)
**ORF12/mK3**	E3 ubiquitin ligase, immune evasion	Immediate early/Late	yes	ΔK3 mutant deficient in the expression or function of mK3	wt	Decreased splenic latency and/or reactivation after i.n.	(26)
**M5**	unknown	Early-late	no	nd	nd	nd	n/a
**M6**	unknown	Immediate early	no	M6.stop mutant deficient in the expression of M6 protein	wt	wt	(27)
**ORF17**	Capsid protein	Late	no	nd	nd	nd	n/a
**ORF18**	Protein UL79, Regulator of late gene expression	Late	no	ORF18.stop mutant deficient in the expression of ORF18 protein	essential	nd	(28)
**ORF19**	DNA packaging tegument protein UL25	Late	no	nd	nd	nd	n/a
**ORF20**	Nuclear protein UL24	Early-late	no	ORF20.stop and ORF20.FR mutants deficient in the expression of ORF20 protein	wt	Severely increased lytic replication in the lungs after i.n.	(29)
**ORF21**	Thymidine kinase (TK)	Early-late	no	ORF21 deletion mutant	wt	Severely decreased lytic replication in the lungs and downstream latency and/or reactivation in the spleen after i.n.; decreased reactivation in the spleen and PECs after i.p.	(30, 31)

**ORF22**	Envelope glycoprotein H (gH), Virion and cell membrane fusion	Late	no	ORF22.stop mutant deficient in the expression of gH	essential	nd	(32, 33)
**ORF23**	Protein UL88	Late	no	ORF23 deletion mutant	wt	wt	(34)
**ORF24**	Virion protein UL87, Regulator of late gene expression	Early	no	ORF24.stop mutant deficient in the expression of ORF24 protein	essential	nd	(35)
**ORF25**	Major capsid protein	Early-late	no	nd	nd	nd	n/a
**ORF26**	Capsid triplex subunit 2	Late	no	nd	nd	nd	n/a
**ORF27**	Envelope glycoprotein 48 (gp48), virus intercellular spread	Late	no	ORF27.stop and ORF27.FRT mutants deficient in the expression of ORF27 protein	wt	Decreased lytic replication in the lungs and downstream splenic latency and/or reactivation after i.n.	(36)
**ORF28**	Envelope glycoprotein	Late	no	ORF28 mutant lacking its transmembrane domain	wt	wt	(37)
**ORF29b**	DNA packaging protein	Late	no	nd	nd	nd	n/a
**ORF30**	Protein UL91, Regulator of late gene expression	Early-late	no	ORF30.stop mutant deficient in the expression of ORF30 protein	essential	nd	(38)
**ORF31**	Protein UL92, regulator of late gene expression	Early-late	no	ORF31.stop mutant deficient in the expression of ORF31 protein	essential	Severely decreased splenic latency establishment after i.n. and i.p.	(39, 40)
**ORF32**	DNA packaging tegument protein UL17	Early-late	no	nd	nd	nd	n/a

**ORF33**	Tegument protein UL16, virion morphogenesis and egress	Late	no	ORF33.stop mutant deficient in the expression of ORF33 protein	essential	nd	(41)
**ORF29a**	DNA packaging protein	Late	no	nd	nd	nd	n/a
**ORF34**	Protein UL95, regulator of late gene expression	Early-late	no	ORF34.stop mutant deficient in the expression of ORF34 protein	essential	nd	(38)
**ORF35**	Tegument protein UL14	Early-late	no	ORF35.stop mutant deficient in the expression of ORF35 protein	↓~5x	Severely decreased lytic replication in the lungs and downstream latency and reactivation in the spleen after i.n.; Severely decreased reactivation in the spleen after i.p.	(42)
**ORF36**	Tegument serine/threonine protein kinase	Early	no	ORF36.stop mutant deficient in the expression of ORF36 protein	↓>10x	Severely decreased lytic replication in the lungs and downstream latency establishment and reactivation in the spleen and PECs after i.n.; severely decreased latency establishment and reactivation in the spleen and PECs after i.p.	(43–46)
				ORF36.KN mutant deficient in the kinase activity of ORF36 protein	↓>100x	Severely decreased lytic replication in the lungs and downstream latency and reactivation in the spleen and PECs after i.n.; severely decreased reactivation in the spleen and PECs after i.p.	(44–46)
**ORF37**	Alkaline exonuclease, SOX homolog, host shutoff	Early-late	no	ORF37.stop mutant deficient in the expression of ORF37 protein	↓>100x	Severely decreased lytic replication in the lungs after i.n.	(47)

				ORF37.ΔHS mutant deficient in the host shutoff activity of ORF37 protein	wt	Severely decreased splenic latency establishment and reactivation after i.n.	(48)
**ORF38**	Myristylated tegument protein	Immediate early	no	nd	nd	nd	n/a
**ORF39**	Envelope glycoprotein M (gM), virion assembly	Late	no	ORF39.stop mutant deficient in the expression of gM	essential	nd	(33)
**ORF40**	Helicase/primase subunit	Early-late	no	nd	nd	nd	n/a
**ORF42**	Tegument protein UL7	Late	no	nd	nd	nd	n/a
**ORF43**	Capsid portal protein	Early-late	no	nd	nd	nd	n/a
**ORF44**	Helicase/primase helicase subunit	Early-late	no	nd	nd	nd	n/a
**ORF45**	Tegument protein G45, virion morphogenesis and egress	Late	no	ORF45.stop mutant deficient in the expression of ORF45 protein	↓>100x	nd	(49, 50)
**ORF46**	Uracil DNA glycosylase (vUNG)	Late	no	ORF46.stop mutant deficient in the expression of ORF46 protein	↓~8x	Severely decreased lytic replication in the lungs and downstream splenic latency establishment and reactivation after i.n.	(51, 52)
				ORF46.CM mutant deficient in the catalytic activity of ORF46	wt	wt	(52)
				ORF46.CM/ORF54.CM mutant deficient in the	wt	Severely decreased lytic replication in the lungs and downstream splenic latency	(52)

				catalytic activity of both ORF46 and ORF54		establishment and reactivation after i.n.	
**ORF47**	Envelope glycoprotein L (gL), Virion and cell membrane fusion	Early-late	no	ORF47.stop and ORF47 deletion mutants deficient in the expression of gL	wt	wt	(32)
**ORF48**	Tegument protein G48	Late	no	ORF48.stop mutant deficient in the expression of ORF48 protein	↓>100x	Decreased lytic replication in the lungs and downstream splenic latency establishment and reactivation after i.n.; decreased splenic latency establishment and reactivation after i.p.	(53)
**ORF49**	Tegument protein G49	Early-late	no	ORF49.stop mutant deficient in the expression of ORF49 protein	↓>10x	Decreased lytic replication in the lungs and downstream splenic latency establishment and reactivation after i.n.	(54, 55)
				F50 mutant carrying two loxP sites flanking ORF49 and ORF50 that enables conditional deletion of ORF49 and ORF50 [i]	essential	Severely decreased splenic latency and/or reactivation after i.n. and i.p in CD19-Cre mice	(56)
**ORF50**	Replication and transcription activator (RTA)	Immediate early	yes	ORF50.stop mutant deficient in the expression of RTA	essential	Undetectable lytic and latent infection in vivo	(57–59)
				G50DblKo mutant lacking both ORF50 distal and proximal promoters	essential	Significantly decreased lytic replication in the lungs and downstream splenic latency establishment and reactivation after i.n.; decreased splenic reactivation after i.p.; decreased latency establishment and reactivation in the PECs after i.p.; decreased lethality in IFNαβR$^{-/-}$ mice	(60)

				F50 mutant carrying two loxP sites flanking ORF49 and ORF50 that enables conditional deletion of ORF49 and ORF50 [i]	essential	Severely decreased splenic latency and/or reactivation after i.n. and i.p in CD19-Cre mice	(56)
**ORF51/M7**	Envelope glycoprotein 150 (gp150), virion release	Late	no	M7.stop mutant deficient in the expression of gp150	defective in release of cell-free virus	Decreased sexual transmission from female to male mice	(61–64)
**ORF52**	Tegument protein G52, virion morphogenesis and egress	Late	no	ORF52.stop mutant deficient in the expression of ORF52 protein	↓>100x	nd	(65–67)
**ORF53**	Envelope glycoprotein N (gN)	Late	no	nd	nd	nd	n/a
**ORF54**	Deoxyuridine triphosphatase (dUTPase)	Early	no	ORF54.stop mutant deficient in the expression of dUTPase	wt	Severely decreased splenic latency establishment after i.n.	(68)
				ORF54.DM mutant deficient in the dUTPase activity	wt	wt (BALB/c)	(68)
				ORF54.CM mutant deficient in the dUTPase activity	nd	Transient decreased lytic replication in the lungs and downstream latency establishment and reactivation in the spleen after i.n. (C57BL/6)	(52)
				ORF46.CM/ORF54.CM mutant deficient in the catalytic activity of both ORF46 and ORF54	wt	Severely decreased lytic replication in the lungs and downstream splenic latency establishment and reactivation after i.n.	(52)
**ORF55**	Tegument protein UL51	Early-late	no	nd	nd	nd	n/a

**ORF56**	Helicase/primase primase subunit	Early-late	no	nd	nd	nd	n/a
**M8**	unknown	Immediate early	yes	nd	nd	nd	n/a
**ORF57**	Multifunctional expression regulator	Immediate early	no	nd	nd	nd	n/a
**vMAP**	Viral mitochondrial anti-apoptotic protein (vMAP)	Immediate early	no	vMAP.stop mutant deficient in the expression of vMAP	↓~10x	nd	(69)
**ORF58**	Tegument protein UL43, cell binding, secondary envelopment	Late	no	ORF58.stop mutant deficient in the expression of ORF58 protein	wt-↓~10x [k]	nd	(70)
**ORF59**	DNA polymerase processivity factor	Late	no	nd	nd	nd	n/a
**ORF60**	Ribonucleotide reductase small subunit protein (RNR-S)	Early-late	no	ORF60.stop and ORF60 deletion mutants deficient in the expression of RNR-S	↓>100x	Decreased lytic replication in the nose and lungs after i.n.; decreased splenic latency maintenance after i.p.	(71)
**ORF61**	Ribonucleotide reductase large subunit protein (RNR-L)	Late	no	ORF61.stop mutant deficient in the expression of RNR-L	↓>100x	Severely decreased lytic replication in the nose and lungs after i.n.	(71, 72)
**ORF62**	Capsid triplex subunit 1	Late	no	nd	nd	nd	n/a
**ORF63**	Tegument protein UL37, capsid trafficking	Early	no	ORF63.stop mutant deficient in the expression of ORF63 protein	↓~10x	Severely decreased lytic replication in the lungs and downstream splenic latency establishment after i.n.	(73)

**ORF64**	Large tegument protein, immune evasion	Early	no	ORF64.C33A mutant deficient in deubiquitinase activity of ORF64	↓~10x	Decreased splenic latency establishment and maintenance after i.n.; decreased acute replication in the spleen after i.p.	(74, 75)
**ORF65/M9**	Small capsid protein	Late	yes	nd	nd	nd	n/a
**ORF66**	Protein UL49	Late	no	nd	nd	nd	n/a
**ORF67**	Nuclear egress type 2 membrane protein	Late	no	ORF67.stop mutant deficient in the expression of ORF67 protein	↓>100x	nd	(76)
**ORF68**	DNA packaging protein UL32	Early	yes	nd	nd	nd	n/a
**ORF69**	Nuclear egress lamina protein	Late	yes	ORF69.stop mutant deficient in the expression of ORF69 protein	↓>100x	nd	(76)
**$M10_{a/b/c}$**	unknown	Early-late	yes	OriLytΔM10ΔTet mutant lacking $M10_{a/b/c}$ coding region	wt	wt	(77)
**ORF72**	v-cyclin, a host cyclin D homolog, Cell cycle regulator	Early-late	yes	ORF72.LacZ, ORF72.stop, and ORF72 deletion mutants deficient in the expression of v-cyclin	wt	Decreased lytic replication in the lungs and downstream splenic reactivation after i.n.; severely decreased reactivation in the spleen and PECs after i.p; decreased LPD in $\beta 2m^{-/-}$ mice	(9, 78–81)
				ORF72.E133V and ORF72.K104E mutants deficient in the binding and activation of cyclin-dependent kinases	wt	Decreased lytic replication in the lungs and downstream splenic reactivation after i.n.	(80)
**M11**	vBcl-2, a host Bcl-2 homolog, anti-apoptotic	Early-late	yes	M11.stop mutant deficient in the expression of vBcl-2	wt	Decreased splenic latency establishment after i.n.; decreased reactivation in the PECs after i.p.; decreased lethality in IFNγR$^{-/-}$ mice; decreased LPD in $\beta 2m^{-/-}$ mice	(9, 82, 83)

**ORF73**	Latency-associated nuclear antigen (mLANA), episome maintenance	Immediate early	yes	ORF73.stop and ORF73 deletion mutants deficient in the expression of mLANA	wt	Decreased lytic replication in the lungs and downstream splenic latency establishment and maintenance, and reactivation after i.n.; decreased splenic latency establishment and reactivation after i.p.; decreased reactivation in the PECs after i.p.	(84–88)
				ORF73.loxP mutant carrying two loxP sites flanking ORF73 that enables conditional deletion of mLANA [i]	wt	Severely decreased splenic latency establishment and reactivation after i.n. and i.p. in CD19-Cre mice	(88)
				ORF73.vSOCS mutant carrying alanine substitutions for SOCS-box-like motif in ORF73 that is deficient in inhibiting NF-kB activity	wt	Severely decreased splenic latency and/or reactivation after i.n.	(89)
				ORF73 V199A, P203A/P206A, and V199A/L202A mutants deficient in E3 ubiquitin ligase activity of mLANA	wt	Decreased splenic latency establishment after i.n.	(90)
**ORF74**	A host G protein coupled receptor homolog (vGPCR)	Early-late	yes	ORF74.stop and ORF74.Δ440 mutants deficient in the expression of vGPCR	wt	Decreased splenic reactivation after i.p.; increased latency maintenance in the PECs after i.p.	(9, 91, 92)
**ORF75C**	Tegument protein G75C	Late	yes	ORF75C.stop mutant deficient in the expression of ORF75C protein	essential	Severely decreased lytic replication in the lungs and downstream splenic latency in the maintenance phase after i.n.	(93, 94)
**ORF75B**	Tegument protein G75B	Early	yes	ORF75B.stop mutant deficient in the expression of ORF75B protein	wt	wt	(95)

**ORF75A**	Tegument protein G75A	Immediate early/Early-late	yes	ORF75A.stop mutant deficient in the expression of ORF75A protein	wt	Decreased lytic replication in the lungs and downstream splenic latency establishment and reactivation after i.n.; decreased splenic reactivation after i.p.	(95)
**M12**	unknown	Early	no	nd	nd	nd	n/a
**M13**	unknown	Immediate early	no	nd	nd	nd	n/a
**M14**	unknown	Immediate early	no	nd	nd	nd	n/a
**Left oriLyt**	Lytic replication	nd	no	Δleft oriLyt mutant lacking the nucleotides 26232-26373 of the genome	wt-↓100x [l]	Decreased lytic replication in the lungs and downstream splenic latency establishment and reactivation after i.n.; decreased latency establishment and reactivation in the PECs after i.p.	(96)
**Right oriLyt**	Lytic replication	nd	no	Δright oriLyt and Δ201nt mutants lacking the nucleotides 101530-101731 of the genome	wt-↓100x [l]	Decreased lytic replication in the lungs and downstream splenic latency establishment and reactivation after i.n.; decreased latency establishment and reactivation in the PECs after i.p.	(77, 96, 97)
**The 40 bp internal repeat**	unknown	nd	no	Delta 40 bp mutant lacking the 40 bp repeat	wt	Decreased splenic latency establishment and reactivation after i.n.	(27)
**The 100 bp internal repeat**	unknown	nd	no	Delta 100 bp mutant lacking the 100 bp repeat	wt	nd	(27)

[a] Abbreviations used in the table: TMER, tRNA-miRNA encoded RNAs; ORF, open reading frame; oriLy, lytic origin of replication; wt, the phenotypes observed resembling those of MHV68 wildtype virus; nd, not determined; ↓, decreased; i.n., intranasal inoculation; i.p. intraperitoneal inoculation; PECs, peritoneal exudate cells; LPD, lymphoproliferative disease; IFNγ$^{-/-}$ mice, interferon γ deficient mice on the BALB/c background; IFNγR$^{-/-}$ mice, interferon γ receptor deficient mice on the 129 background; β2m$^{-/-}$ mice, β2m deficient mice on the BALB/c background; CD1 mice, deficient in both Cd1d1 and Cd1d2 genes on the 129 background; F1 mice, F1 deficient mice on a mixed 129 x B6 background; Factor B$^{-/-}$ mice, Factor B deficient mice; Rag1$^{-/-}$ mice, Rag1 deficient mice on the C57BL/6J background; IFNαβR$^{-/-}$ mice, Interferon α and β receptor deficient mice on a mixed 129S2/SvPas. background; n/a, not applicable.

[b] Functional annotations are based on the previous publications (59, 69, 98, 99).

[c] Kinetic classifications are based on the previous publications (69, 98, 100).
[d] Determination of latent expression of specific genes is based on the previous publications (3, 69, 100–103).
[e] A concise description for MHV68 mutant viruses is indicated. Refer to the corresponding references for detailed mutant virus descriptions if needed.
[f] If the phenotypes were near or below limit of detection, "essential" is indicated.
[g] The data from mutant viruses using transposon mutagenesis approaches are not used here to evaluate the requirement for a specific viral gene during lytic replication in vitro, but they are available from the previous studies (104, 105).
[h] If the phenotype difference is ≥100x, "severely decreased" or "severely increased" is indicated, otherwise "decreased" or "increased" is indicated.
[i] Indicates that the phenotypes were observed in CD19-Cre mice.
[j] Indicates that the phenotypes were observed in wood mice, a natural host of MHV68.
[k] The lytic replication phenotypes observed for the ORF58.stop mutant virus were dependent on multiplicity of infection.
[l] Indicates that the in vitro lytic replication phenotypes observed were cell-type dependent.

LITERATURE CITED

1. Diebel KW, Oko LM, Medina EM, Niemeyer BF, Warren CJ, et al. 2015. Gammaherpesvirus small noncoding RNAs are bifunctional elements that regulate infection and contribute to virulence in vivo. *MBio*. 6(1):e01670-14

2. Steer B, Strehle M, Sattler C, Bund D, Flach B, et al. 2016. The small noncoding RNAs (sncRNAs) of murine gammaherpesvirus 68 (MHV-68) are involved in regulating the latent-to-lytic switch in vivo. *Sci. Rep.* 6:32128

3. Feldman ER, Kara M, Coleman CB, Grau KR, Oko LM, et al. 2014. Virus-encoded microRNAs facilitate gammaherpesvirus latency and pathogenesis in vivo. *MBio*. 5(3):e00981-14

4. Feldman ER, Kara M, Oko LM, Grau KR, Krueger BJ, et al. 2016. A gammaherpesvirus noncoding RNA is essential for hematogenous dissemination and establishment of peripheral latency. *mSphere*. 1(2):e00105-15

5. Wang Y, Feldman ER, Bullard WL, Tibbetts SA. 2019. A gammaherpesvirus microRNA targets EWSR1 (Ewing sarcoma breakpoint region 1) in vivo to promote latent infection of germinal center B cells. *MBio*. 10(4):e00996-19

6. Kara M, O'Grady T, Feldman ER, Feswick A, Wang Y, et al. 2019. Gammaherpesvirus readthrough transcription generates a long non-coding RNA that is regulated by antisense miRNAs and correlates with enhanced lytic replication in vivo. *Non-coding RNA*. 5(1):6

7. Clambey ET, Virgin HW 4th, Speck SH. 2000. Disruption of the murine gammaherpesvirus 68 M1 open reading frame leads to enhanced reactivation from latency. *J. Virol.* 74(4):1973–84

8. Evans AG, Moser JM, Krug LT, Pozharskaya V, Mora AL, Speck SH. 2008. A gammaherpesvirus-secreted activator of Vβ4+ CD8+ T cells regulates chronic infection and immunopathology. *J. Exp. Med.* 205(3):669–84

9. Tarakanova VL, Kreisel F, White DW, Virgin HW 4th. 2008. Murine gammaherpesvirus 68 genes both induce and suppress lymphoproliferative disease. *J. Virol.* 82(2):1034–39

10. Krug LT, Evans AG, Gargano LM, Paden CR, Speck SH. 2013. The absence of M1 leads to increased establishment of murine gammaherpesvirus 68 latency in IgD-negative B cells. *J. Virol.* 87(6):3597–3604

11. Jacoby MA, Virgin HW 4th, Speck SH. 2002. Disruption of the M2 gene of murine gammaherpesvirus 68 alters splenic latency following intranasal, but not intraperitoneal, inoculation. *J. Virol.* 76(4):1790–1801

12. Herskowitz J, Jacoby MA, Speck SH. 2005. The murine gammaherpesvirus 68 M2 gene is required for efficient reactivation from latently infected B cells. *J. Virol.* 79(4):2261–73

13. Owens SM, Oldenburg DG, White DW, Forrest JC. 2020. Deletion of murine gammaherpesvirus gene M2 in activation-induced cytidine deaminase-expressing B cells impairs host colonization and viral reactivation. *J. Virol.* 95(1):e01933-20

14. Macrae AI, Usherwood EJ, Husain SM, Flaño E, Kim I-J, et al. 2003. Murid herpesvirus 4 strain 68 M2 protein is a B-cell-associated antigen important for latency but not lymphocytosis. *J. Virol.* 77(17):9700–9709

15. Simas JP, Marques S, Bridgeman A, Efstathiou S, Adler H. 2004. The M2 gene product of murine gammaherpesvirus 68 is required for efficient colonization of splenic follicles but is not necessary for expansion of latently infected germinal centre B cells. *J. Gen. Virol.* 85(10):2789–97

16. Herskowitz JH, Siegel AM, Jacoby MA, Speck SH. 2008. Systematic mutagenesis of the murine gammaherpesvirus 68 M2 protein identifies domains important for chronic infection. *J. Virol.* 82(7):3295–3310

17. van Berkel V, Levine B, Kapadia SB, Goldman JE, Speck SH, Virgin HW 4th. 2002. Critical role for a high-affinity chemokine-binding protein in gamma-herpesvirus-induced lethal meningitis. *J. Clin. Invest.* 109(7):905–14

18. Hughes DJ, Kipar A, Leeming GH, Bennett E, Howarth D, et al. 2011. Chemokine binding protein M3 of murine gammaherpesvirus 68 modulates the host response to infection in a natural host. *PLoS Pathog.* 7(3):e1001321

19. Evans AG, Moorman NJ, Willer DO, Speck SH. 2006. The M4 gene of γHV68 encodes a secreted glycoprotein and is required for the efficient establishment of splenic latency. *Virology*. 344(2):520–31

20. Kapadia SB, Levine B, Speck SH, Virgin HW 4th. 2002. Critical role of complement and viral evasion of complement in acute, persistent, and latent gamma-herpesvirus infection. *Immunity*. 17(2):143–55

21. Adler H, Messerle M, Wagner M, Koszinowski UH. 2000. Cloning and mutagenesis of the murine gammaherpesvirus 68 genome as an infectious bacterial artificial chromosome. *J. Virol.* 74(15):6964–74

22. Gillet L, May JS, Stevenson PG. 2009. In vivo importance of heparan sulfate-binding glycoproteins for murid herpesvirus-4 infection. *J. Gen. Virol.* 90(3):602–13

23. Tibbetts SA, Suarez F, Steed AL, Simmons JA, Virgin HW 4th. 2006. A gamma-herpesvirus deficient in replication establishes chronic infection in vivo and is

impervious to restriction by adaptive immune cells. *Virology*. 353(1):210–19

24. Glauser DL, Milho R, Frederico B, May JS, Kratz A-S, et al. 2013. Glycoprotein B cleavage is important for murid herpesvirus 4 to infect myeloid cells. *J. Virol.* 87(19):10828–42

25. Boname JM, May JS, Stevenson PG. 2005. Murine gammaherpesvirus 68 open reading frame 11 encodes a nonessential virion component. *J. Virol.* 79(5):3163–68

26. Stevenson PG, May JS, Smith XG, Marques S, Adler H, et al. 2002. K3-mediated evasion of CD8(+) T cells aids amplification of a latent gamma-herpesvirus. *Nat. Immunol.* 3(8):733–40

27. Thakur NN, El-Gogo S, Steer B, Freimüller K, Waha A, Adler H. 2007. A gammaherpesviral internal repeat contributes to latency amplification. *PLoS One*. 2(8):e733

28. Arumugaswami V, Wu T-T, Martinez-Guzman D, Jia Q, Deng H, et al. 2006. ORF18 is a transfactor that is essential for late gene transcription of a gammaherpesvirus. *J. Virol.* 80(19):9730–40

29. Nascimento R, Costa H, Dias JD, Parkhouse RME. 2011. MHV-68 open reading frame 20 is a nonessential gene delaying lung viral clearance. *Arch. Virol.* 156(3):375–86

30. Coleman HM, de Lima B, Morton V, Stevenson PG. 2003. Murine gammaherpesvirus 68 lacking thymidine kinase shows severe attenuation of lytic cycle replication in vivo but still establishes latency. *J. Virol.* 77(4):2410–17

31. Gill MB, Wright DE, Smith CM, May JS, Stevenson PG. 2009. Murid herpesvirus-4 lacking thymidine kinase reveals route-dependent requirements for host colonization. *J. Gen. Virol.* 90(6):1461–70

32. Gillet L, May JS, Colaco S, Stevenson PG. 2007. Glycoprotein L disruption reveals two functional forms of the murine gammaherpesvirus 68 glycoprotein H. *J. Virol.* 81(1):280–91

33. May JS, Colaco S, Stevenson PG. 2005. Glycoprotein M is an essential lytic replication protein of the murine gammaherpesvirus 68. *J. Virol.* 79(6):3459–67

34. Ohno S, Steer B, Sattler C, Adler H. 2012. ORF23 of murine gammaherpesvirus 68 is non-essential for in vitro and in vivo infection. *J. Gen. Virol.* 93(5):1076–80

35. Wong E, Wu T-T, Reyes N, Deng H, Sun R. 2007. Murine gammaherpesvirus 68 open reading frame 24 is required for late gene expression after DNA replication. *J. Virol.* 81(12):6761–64

36. May JS, Walker J, Colaco S, Stevenson PG. 2005. The murine gammaherpesvirus 68 ORF27 gene product contributes to intercellular viral spread. *J. Virol.* 79(8):5059–68

37. May JS, Coleman HM, Boname JM, Stevenson PG. 2005. Murine gammaherpesvirus-68 ORF28 encodes a non-essential virion glycoprotein. *J. Gen. Virol.* 86(4):919–28

38. Wu T-T, Park T, Kim H, Tran T, Tong L, et al. 2009. ORF30 and ORF34 are essential for expression of late genes in murine gammaherpesvirus 68. *J. Virol.*

83(5):2265–73

39. Jia Q, Wu T-T, Liao H-I, Chernishof V, Sun R. 2004. Murine gammaherpesvirus 68 open reading frame 31 is required for viral replication. *J. Virol.* 78(12):6610–20

40. Flaño E, Jia Q, Moore J, Woodland DL, Sun R, Blackman MA. 2005. Early establishment of gamma-herpesvirus latency: implications for immune control. *J. Immunol.* 174(8):4972–78

41. Guo H, Wang L, Peng L, Zhou ZH, Deng H. 2009. Open reading frame 33 of a gammaherpesvirus encodes a tegument protein essential for virion morphogenesis and egress. *J. Virol.* 83(20):10582–95

42. Hikita S-I, Yanagi Y, Ohno S. 2015. Murine gammaherpesvirus 68 ORF35 is required for efficient lytic replication and latency. *J. Gen. Virol.* 96(12):3624–34

43. Tarakanova VL, Leung-Pineda V, Hwang S, Yang C-W, Matatall K, et al. 2007. Gamma-herpesvirus kinase actively initiates a DNA damage response by inducing phosphorylation of H2AX to foster viral replication. *Cell Host Microbe*. 1(4):275–86

44. Hwang S, Kim KS, Flano E, Wu T-T, Tong LM, et al. 2009. Conserved herpesviral kinase promotes viral persistence by inhibiting the IRF-3-mediated type I interferon response. *Cell Host Microbe*. 5(2):166–78

45. Tarakanova VL, Stanitsa E, Leonardo SM, Bigley TM, Gauld SB. 2010. Conserved gammaherpesvirus kinase and histone variant H2AX facilitate gammaherpesvirus latency in vivo. *Virology*. 405(1):50–61

46. Darrah EJ, Jondle CN, Johnson KE, Xin G, Lange PT, et al. 2019. Conserved gammaherpesvirus protein kinase selectively promotes irrelevant B cell responses. *J. Virol.* 93(8):e01760-18

47. Sheridan V, Polychronopoulos L, Dutia BM, Ebrahimi B. 2014. A shutoff and exonuclease mutant of murine gammaherpesvirus-68 yields infectious virus and causes RNA loss in type I interferon receptor knockout cells. *J. Gen. Virol.* 95(5):1135–43

48. Richner JM, Clyde K, Pezda AC, Cheng BYH, Wang T, et al. 2011. Global mRNA degradation during lytic gammaherpesvirus infection contributes to establishment of viral latency. *PLoS Pathog.* 7(7):e1002150

49. Jia X, Shen S, Lv Y, Zhang Z, Guo H, Deng H. 2016. Tegument protein ORF45 plays an essential role in virion morphogenesis of murine gammaherpesvirus 68. *J. Virol.* 90(16):7587–92

50. Jia Q, Chernishof V, Bortz E, Mchardy I, Wu T-T, et al. 2005. Murine gammaherpesvirus 68 open reading frame 45 plays an essential role during the immediate-early phase of viral replication. *J. Virol.* 79(8):5129–41

51. Minkah N, Macaluso M, Oldenburg DG, Paden CR, White DW, et al. 2015. Absence of the uracil DNA glycosylase of murine gammaherpesvirus 68 impairs replication and delays the establishment of latency in vivo. *J. Virol.* 89(6):3366–79

52. Dong Q, Smith KR, Oldenburg DG, Shapiro M, Schutt WR, et al. 2018. Combinatorial loss of the enzymatic activities of viral uracil-DNA glycosylase and viral dUTPase impairs murine gammaherpesvirus pathogenesis and leads to increased recombination-based deletion in the viral genome. *MBio*. 9(5):e01831-18

53. Qi J, Han C, Gong D, Liu P, Zhou S, Deng H. 2015. Murine gammaherpesvirus 68 ORF48 is an RTA-responsive gene product and functions in both viral lytic replication and latency during in vivo infection. *J. Virol.* 89(11):5788–5800

54. Lee S, Cho H-J, Park J-J, Kim Y-S, Hwang S, et al. 2007. The ORF49 protein of murine gammaherpesvirus 68 cooperates with RTA in regulating virus replication. *J. Virol.* 81(18):9870–77

55. Noh C-W, Cho H-J, Kang H-R, Jin HY, Lee S, et al. 2012. The virion-associated open reading frame 49 of murine gammaherpesvirus 68 promotes viral replication both in vitro and in vivo as a derepressor of RTA. *J. Virol.* 86(2):1109–18

56. Lawler C, de Miranda MP, May J, Wyer O, Simas JP, Stevenson PG. 2018. Gammaherpesvirus colonization of the spleen requires lytic replication in B cells. *J. Virol.* 92(7):e02199-17

57. Pavlova I V, Virgin HW 4th, Speck SH. 2003. Disruption of gammaherpesvirus 68 gene 50 demonstrates that Rta is essential for virus replication. *J. Virol.* 77(10):5731–39

58. Moser JM, Farrell ML, Krug LT, Upton JW, Speck SH. 2006. A gammaherpesvirus 68 gene 50 null mutant establishes long-term latency in the lung but fails to vaccinate against a wild-type virus challenge. *J. Virol.* 80(3):1592–98

59. Barton E, Mandal P, Speck SH. 2011. Pathogenesis and host control of gammaherpesviruses: lessons from the mouse. *Annu. Rev. Immunol.* 29:351–97

60. Wakeman BS, Johnson LS, Paden CR, Gray KS, Virgin HW 4th, Speck SH. 2014. Identification of alternative transcripts encoding the essential murine gammaherpesvirus lytic transactivator RTA. *J. Virol.* 88(10):5474–90

61. Stewart JP, Janjua NJ, Pepper SD, Bennion G, Mackett M, et al. 1996. Identification and characterization of murine gammaherpesvirus 68 gp150: a virion membrane glycoprotein. *J. Virol.* 70(6):3528–35

62. de Lima BD, May JS, Stevenson PG. 2004. Murine gammaherpesvirus 68 lacking gp150 shows defective virion release but establishes normal latency in vivo. *J. Virol.* 78(10):5103–12

63. Ruiss R, Ohno S, Steer B, Zeidler R, Adler H. 2012. Murine gammaherpesvirus 68 glycoprotein 150 does not contribute to latency amplification in vivo. *Virol. J.* 9:107

64. Zeippen C, Javaux J, Xiao X, Ledecq M, Mast J, et al. 2017. The major envelope glycoprotein of murid herpesvirus 4 promotes sexual transmission. *J. Virol.* 91(13):e00235-17

65. Bortz E, Wang L, Jia Q, Wu T-T, Whitelegge JP, et al. 2007. Murine gammaherpesvirus 68 ORF52 encodes a tegument protein required for virion morphogenesis in the cytoplasm. *J. Virol.* 81(18):10137–50

66. Benach J, Wang L, Chen Y, Ho CK, Lee S, et al. 2007. Structural and functional studies of the abundant tegument protein ORF52 from murine gammaherpesvirus 68. *J. Biol. Chem.* 282(43):31534–41

67. Wang L, Guo H, Reyes N, Lee S, Bortz E, et al. 2012. Distinct domains in ORF52 tegument protein mediate essential functions in murine gammaherpesvirus 68 virion tegumentation and secondary envelopment. *J. Virol.* 86(3):1348–57

68. Leang RS, Wu T-T, Hwang S, Liang LT, Tong L, et al. 2011. The anti-interferon activity of conserved viral dUTPase ORF54 is essential for an effective MHV-68 infection. *PLoS Pathog.* 7(10):e1002292

69. Feng P, Liang C, Shin YC, Xiaofei E, Zhang W, et al. 2007. A novel inhibitory mechanism of mitochondrion-dependent apoptosis by a herpesviral protein. *PLoS Pathog.* 3(12):e174

70. May JS, de Lima BD, Colaco S, Stevenson PG. 2005. Intercellular gamma-herpesvirus dissemination involves co-ordinated intracellular membrane protein transport. *Traffic*. 6(9):780–93

71. Milho R, Gill MB, May JS, Colaco S, Stevenson PG. 2011. In vivo function of the murid herpesvirus-4 ribonucleotide reductase small subunit. *J. Gen. Virol.* 92(7):1550–60

72. Gill MB, May JS, Colaco S, Stevenson PG. 2010. Important role for the murid herpesvirus 4 ribonucleotide reductase large subunit in host colonization via the respiratory tract. *J. Virol.* 84(20):10937–42

73. Latif MB, Machiels B, Xiao X, Mast J, Vanderplasschen A, Gillet L. 2015. Deletion of murid herpesvirus 4 ORF63 affects the trafficking of incoming capsids toward the nucleus. *J. Virol.* 90(5):2455–72

74. Gredmark-Russ S, Isaacson MK, Kattenhorn L, Cheung EJ, Watson N, Ploegh HL. 2009. A gammaherpesvirus ubiquitin-specific protease is involved in the establishment of murine gammaherpesvirus 68 infection. *J. Virol.* 83(20):10644–52

75. Sun C, Schattgen SA, Pisitkun P, Jorgensen JP, Hilterbrand AT, et al. 2015. Evasion of innate cytosolic DNA sensing by a gammaherpesvirus facilitates establishment of latent infection. *J. Immunol.* 194(4):1819–31

76. Lv Y, Shen S, Xiang L, Jia X, Hou Y, et al. 2019. Functional Identification and characterization of the nuclear egress complex of a gammaherpesvirus. *J. Virol.* 93(24):e01422-19

77. Flach B, Steer B, Thakur NN, Haas J, Adler H. 2009. The M10 locus of murine gammaherpesvirus 68 contributes to both the lytic and the latent phases of infection. *J. Virol.* 83(16):8163–72

78. Hoge AT, Hendrickson SB, Burns WH. 2000. Murine gammaherpesvirus 68 cyclin D homologue is required for efficient reactivation from latency. *J. Virol.* 74(15):7016–23

79. van Dyk LF, Virgin HW 4th, Speck SH. 2000. The murine gammaherpesvirus 68 v-cyclin is a critical regulator of reactivation from latency. *J. Virol.* 74(16):7451–61

80. Upton JW, Speck SH. 2006. Evidence for CDK-dependent and CDK-independent functions of the murine gammaherpesvirus 68 v-cyclin. *J. Virol.* 80(24):11946–59

81. Williams LM, Niemeyer BF, Franklin DS, Clambey ET, van Dyk LF. 2015. A conserved gammaherpesvirus cyclin specifically bypasses host p18INK4c to promote reactivation from latency. *J. Virol.* 89(21):10821–31

82. Gangappa S, van Dyk LF, Jewett TJ, Speck SH, Virgin HW 4th. 2002. Identification of the in vivo role of a viral bcl-2. *J. Exp. Med.* 195(7):931–40

83. de Lima BD, May JS, Marques S, Simas JP, Stevenson PG. 2005. Murine gammaherpesvirus 68 bcl-2 homologue contributes to latency establishment in vivo. *J. Gen. Virol.* 86(1):31–40

84. Moorman NJ, Willer DO, Speck SH. 2003. The gammaherpesvirus 68 latency-associated nuclear antigen homolog is critical for the establishment of splenic latency. *J. Virol.* 77(19):10295–303

85. Fowler P, Marques S, Simas JP, Efstathiou S. 2003. ORF73 of murine herpesvirus-68 is critical for the establishment and maintenance of latency. *J. Gen. Virol.* 84(12):3405–16

86. Forrest JC, Paden CR, Allen RD 3rd, Collins J, Speck SH. 2007. ORF73-null murine gammaherpesvirus 68 reveals roles for mLANA and p53 in virus replication. *J. Virol.* 81(21):11957–71

87. Paden CR, Forrest JC, Moorman NJ, Speck SH. 2010. Murine gammaherpesvirus 68 LANA is essential for virus reactivation from splenocytes but not long-term carriage of viral genome. *J. Virol.* 84(14):7214–24

88. Salinas E, Gupta A, Sifford JM, Oldenburg DG, White DW, Forrest JC. 2018. Conditional mutagenesis in vivo reveals cell type- and infection stage-specific requirements for LANA in chronic MHV68 infection. *PLoS Pathog.* 14(1):e1006865

89. Rodrigues L, Filipe J, Seldon MP, Fonseca L, Anrather J, et al. 2009. Termination of NF-kappaB activity through a gammaherpesvirus protein that assembles an EC5S ubiquitin-ligase. *EMBO J.* 28(9):1283–95

90. Cerqueira SA, Tan M, Li S, Juillard F, McVey CE, et al. 2016. Latency-associated nuclear antigen E3 ubiquitin ligase activity impacts gammaherpesvirus-driven germinal center B cell proliferation. *J. Virol.* 90(17):7667–83

91. Moorman NJ, Virgin HW 4th, Speck SH. 2003. Disruption of the gene encoding the γHV68 v-GPCR leads to decreased efficiency of reactivation from latency. *Virology*. 307(2):179–90

92. Lee BJ, Koszinowski UH, Sarawar SR, Adler H. 2003. A gammaherpesvirus G protein-coupled receptor homologue is required for increased viral replication in response to chemokines and efficient reactivation from latency. *J. Immunol.* 170(1):243–51

93. Ling PD, Tan J, Sewatanon J, Peng R. 2008. Murine gammaherpesvirus 68 open reading frame 75c tegument protein induces the degradation of PML and is essential for production of infectious virus. *J. Virol.* 82(16):8000–8012

94. Gaspar M, Gill MB, Lösing J-B, May JS, Stevenson PG. 2008. Multiple functions for ORF75c in murid herpesvirus-4 infection. *PLoS One*. 3(7):e2781

95. Van Skike ND, Minkah NK, Hogan CH, Wu G, Benziger PT, et al. 2018. Viral FGARAT ORF75A promotes early events in lytic infection and gammaherpesvirus pathogenesis in mice. *PLoS Pathog.* 14(2):e1006843

96. Sattler C, Steer B, Adler H. 2016. Multiple lytic origins of replication are required for optimal gammaherpesvirus fitness in vitro and in vivo. *PLoS Pathog.* 12(3):e1005510

97. Adler H, Steer B, Freimüller K, Haas J. 2007. Murine gammaherpesvirus 68 contains two functional lytic origins of replication. *J. Virol.* 81(13):7300–7305

98. Cheng BYH, Zhi J, Santana A, Khan S, Salinas E, et al. 2012. Tiled microarray identification of novel viral transcript structures and distinct transcriptional

profiles during two modes of productive murine gammaherpesvirus 68 infection. *J. Virol.* 86(8):4340–57

99. Virgin HW 4th, Latreille P, Wamsley P, Hallsworth K, Weck KE, et al. 1997. Complete sequence and genomic analysis of murine gammaherpesvirus 68. *J. Virol.* 71(8):5894–5904

100. O'Grady T, Feswick A, Hoffman BA, Wang Y, Medina EM, et al. 2019. Genome-wide transcript structure resolution reveals abundant alternate isoform usage from murine gammaherpesvirus 68. *Cell Rep.* 27(13):3988-4002.e5

101. Virgin HW 4th, Presti RM, Li X-Y, Liu C, Speck SH. 1999. Three distinct regions of the murine gammaherpesvirus 68 genome are transcriptionally active in latently infected mice. *J. Virol.* 73(3):2321–32

102. Simas JP, Swann D, Bowden R, Efstathiou S. 1999. Analysis of murine gammaherpesvirus-68 transcription during lytic and latent infection. *J. Gen. Virol.* 80:75–82

103. Martinez-Guzman D, Rickabaugh T, Wu T-T, Brown H, Cole S, et al. 2003. Transcription program of murine gammaherpesvirus 68. *J. Virol.* 77(19):10488–503

104. Moorman NJ, Lin CY, Speck SH. 2004. Identification of candidate gammaherpesvirus 68 genes required for virus replication by signature-tagged transposon mutagenesis. *J. Virol.* 78(19):10282–90

105. Song MJ, Hwang S, Wong WH, Wu T-T, Lee S, et al. 2005. Identification of viral genes essential for replication of murine gamma-herpesvirus 68 using signature-tagged mutagenesis. *Proc. Natl. Acad. Sci. U. S. A.* 102(10):3805–10
