# Supplementary material for: Conquering the Host: Determinants of Pathogenesis Learned from Murine Gammaherpesvirus 68
Source: Annu Rev Virol. Author manuscript; Available in PMC 2022 May 31. (PMC9153731; doi:10.1146/annurev-virology-011921-082615)
Supplement: Supp Figs 1-3 [file NIHMS1807025-supplement-Supp_Figs_1-3.pdf]

Supplemental Material: Annu. Rev. Virol. 2021. 8:349-371
https://doi.org/10.1146/annurev-virology-011921-082615
*Conquering the Host: Determinants of Pathogenesis Learned from Murine Gammaherpesvirus 68*
Wang, Tibbetts, and Krug

**Supplemental Figure 1. MHV68 infection of multiple B cell subsets and participation in the germinal center reaction.**
① MHV68 infects naïve B cells and enters the germinal center reactions ② to undergo clonal expansion and somatic hypermutation of the immunoglobulin genes as centroblasts in the dark zone, followed ③ by rounds of selection and immunoglobulin class switching via interactions with follicular dendritic cells and IL-21 producing CD4+ T helper cells in the light zone. Reiterative cyclical rounds of these processes are possible prior to differentiation and exit. ④ MHV68 infected B cells ultimately exit as isotype class-switched memory B cells or plasma cells. Plasmablasts are a source of reactivation at the peak of splenic latency. Created with Biorender.com.

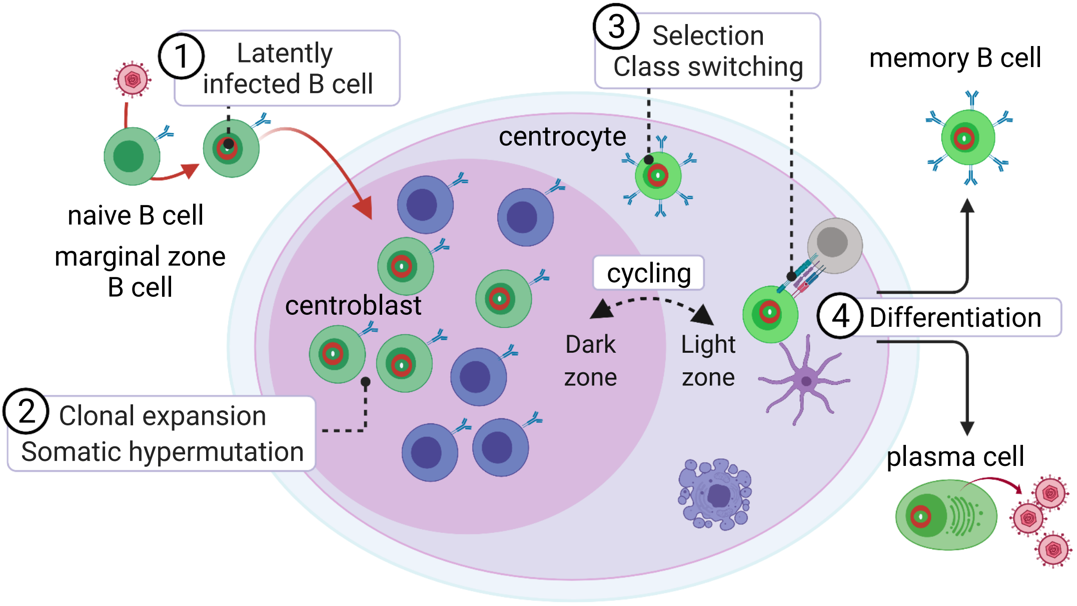

1 Latently infected B cell
naive B cell
marginal zone B cell
2 Clonal expansion Somatic hypermutation
centroblast
centrocyte
3 Selection Class switching
cycling
Dark zone
Light zone
memory B cell
4 Differentiation
plasma cell

**Supplemental Figure 2. Host immune response that controls MHV68 infected cells.** CD8+ T cells control productive lytic infection in epithelial cells in addition to latency in macrophages and B cells via antiviral cytokines and effector CTL responses. CD4+ T cells control infected alveolar macrophages via the recruitment of natural killer cells. CD4+ T cells provide helper functions to CD8+ T cells via IL-2 and promote B cell latency via CD40L and IL-21 in the germinal center. IL-10 is a regulatory cytokine that suppresses CD4+ and CD8+ T cells while promoting B cell proliferation. Interferon (IFN) α, β, and λ (1) cytokines are key to controlling productive replication while IFNγ prevents reactivation of MHV68 from latent B cells and macrophages. Created with Biorender.com.

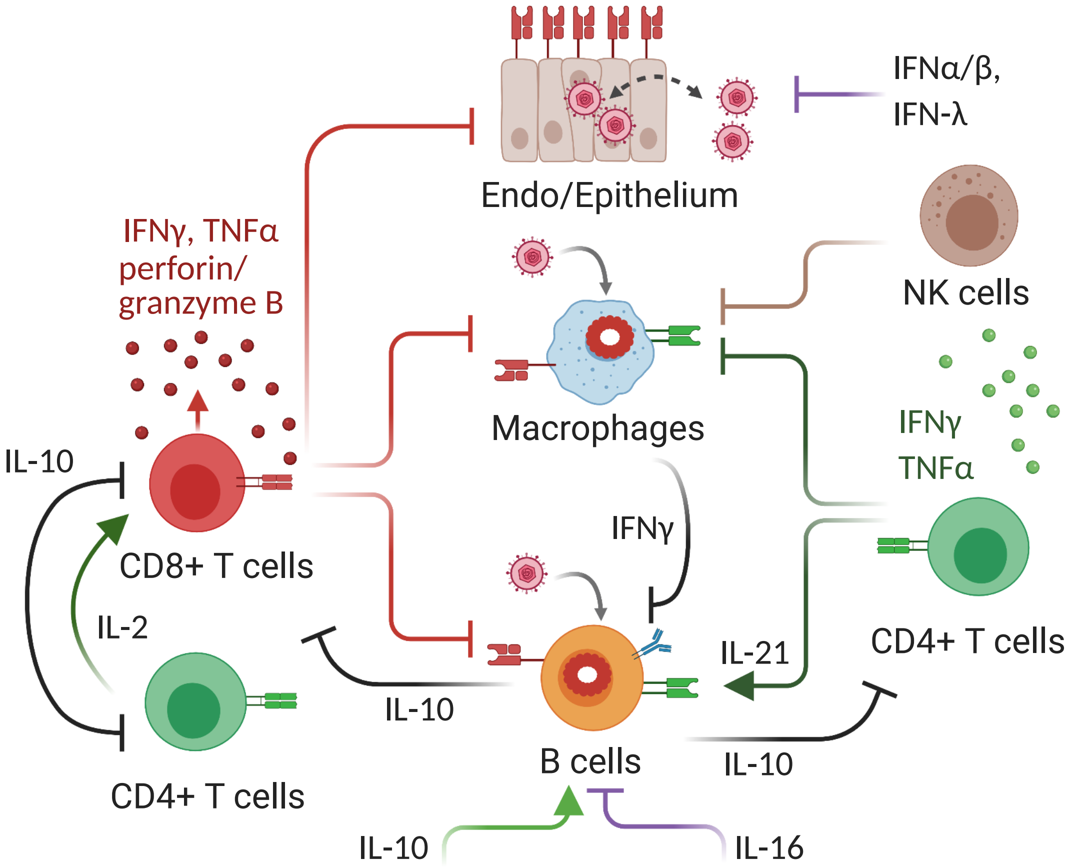

Endo/Epithelium
IFNα/β,
IFN-λ
IFNγ, TNFα
perforin/
granzyme B
Macrophages
NK cells
IFNγ
TNFα
IL-10
CD8+ T cells
IFNγ
IL-2
IL-21
CD4+ T cells
IL-10
CD4+ T cells
B cells
IL-10
IL-10
IL-16

**Supplemental Figure 3. Co-Infection of MHV68 with other pathogens leads to differential outcomes of disease.**

(A) Latent MHV68 infection typically induces an inflammatory milieu such as IFNγ that is protective against numerous bacterial pathogens including *Listeria monocytogenes*, *Yersenia pestis*, *Mycobacterium tuberculosis* and influenza virus (2–5). (B) MHV68 infection of mice 15 and 7 days prior to infection with malarial pathogen *Plasmodium yoelii* XNL leads to 100% lethality due to a suppression of anti-malarial antibody responses (6). (C) Helminth infection following MHV68 infection leads to an induction of IL-4 that promotes RTA expression and MHV68 reactivation (7). (D) Infection with helminths 22 days prior to intranasal infection with MHV68 reduces acute lung replication via virus-specific effector CD8+ T cell responses (8). Created with Biorender.com.

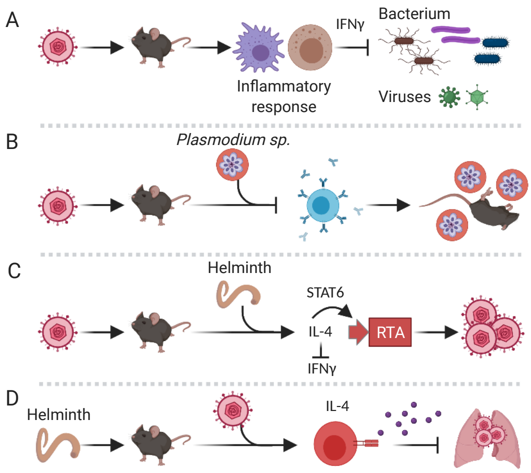
A
IFNγ
Bacterium
Inflammatory response
Viruses
B
*Plasmodium sp.*
C
Helminth
STAT6
IL-4
RTA
IFNγ
D
Helminth
IL-4

**LITERATURE CITED**


1. Jacobs S, Zeippen C, Wavreil F, Gillet L, Michiels T. 2019. IFN-λ decreases murid herpesvirus-4 infection of the olfactory epithelium but fails to prevent virus reactivation in the vaginal mucosa. *Viruses*. 11(8):757
2. Reese TA. 2016. Coinfections: another variable in the herpesvirus latency-reactivation dynamic. *J. Virol.* 90(12):5534–37
3. Barton ES, White DW, Cathelyn JS, Brett-McClellan KA, Engle M, et al. 2007. Herpesvirus latency confers symbiotic protection from bacterial infection. *Nature*. 447(7142):326–29
4. Miller HE, Johnson KE, Tarakanova VL, Robinson RT. 2019. γ-herpesvirus latency attenuates Mycobacterium tuberculosis infection in mice. *Tuberculosis*. 116:56–60
5. Saito F, Ito T, Connett JM, Schaller MA, Carson WF 4th, et al. 2013. MHV68 latency modulates the host immune response to influenza A virus. *Inflammation*. 36(6):1295–1303
6. Matar CG, Anthony NR, O'Flaherty BM, Jacobs NT, Priyamvada L, et al. 2015. Gammaherpesvirus co-infection with malaria suppresses anti-parasitic humoral immunity. *PLoS Pathog.* 11(5):e1004858
7. Reese TA, Wakeman BS, Choi HS, Hufford MM, Huang SC, et al. 2014. Helminth infection reactivates latent γ-herpesvirus via cytokine competition at a viral promoter. *Science*. 345(6196):573–77
8. Rolot M, Dougall AM, Chetty A, Javaux J, Chen T, et al. 2018. Helminth-induced IL-4 expands bystander memory CD8(+) T cells for early control of viral infection. *Nat. Commun.* 9(1):4516
